# CRISPR-Cas9-mediated deletions of *FvMYB46* in *Fragaria vesca* reveal its role in regulation of fruit set and phenylpropanoid biosynthesis

**DOI:** 10.1186/s12870-024-06041-0

**Published:** 2025-02-25

**Authors:** Arti Rai, Magne Nordang Skårn, Abdelhameed Elameen, Torstein Tengs, Mathias Rudolf Amundsen, Oskar S. Bjorå, Lisa K. Haugland, Igor A. Yakovlev, May Bente Brurberg, Tage Thorstensen

**Affiliations:** 1 Division of Biotechnology and Plant Health, Norwegian Institute of Bioeconomy Research, Ås, Norway; 2 Department of Plant Sciences, Norwegian University of Life Sciences, Ås, Norway; 3 Faculty of Environmental Sciences and Natural Resource Management, Norwegian University of Life Sciences, Ås, Norway

**Keywords:** FvMYB46, CRISPR-Cas9, Fruit set, Transcriptomics, Strawberry, Phenylpropanoid, Flavonoid

## Abstract

The phenylpropanoid pathway, regulated by transcription factors of the MYB family, produces secondary metabolites that play important roles in fertilization and early phase of fruit development. The MYB46 transcription factor is a key regulator of secondary cell wall structure, lignin and flavonoid biosynthesis in many plants, but little is known about its activity in flowers and berries in *F. vesca*. For functional analysis of FvMYB46, we designed a CRISPR-Cas9 construct with an endogenous *F. vesca*-specific U6 promoter for efficient and specific expression of two gRNAs targeting the first exon of *FvMYB46*. This generated mutants with an in-frame 81-bp deletion of the first conserved MYB domain or an out-of-frame 82-bp deletion potentially knocking out gene function. In both types of mutant plants, pollen germination and fruit set were significantly reduced compared to wild type. Transcriptomic analysis of flowers revealed that FvMYB46 positively regulates the expression of genes involved in processes like xylan biosynthesis and metabolism, homeostasis of reactive oxygen species (ROS) and the phenylpropanoid pathway, including secondary cell wall biosynthesis and flavonoid biosynthesis. Genes regulating carbohydrate metabolism and signalling were also deregulated, suggesting that FvMYB46 might regulate the crosstalk between carbohydrate metabolism and phenylpropanoid biosynthesis. In the *FvMYB46*-mutant flowers, the flavanol and flavan-3-ol contents, especially epicatechin, quercetin-glucoside and kaempferol-3-coumaroylhexoside, were reduced, and we observed a local reduction in the lignin content in the anthers. Together, these results suggest that FvMYB46 controls fertility and efficient fruit set by regulating the cell wall structure, flavonoid biosynthesis, carbohydrate metabolism, and sugar and ROS signalling in flowers and early fruit development in *F. vesca*.

**Supplementary Information:**

The online version contains supplementary material available at 10.1186/s12870-024-06041-0.

## Background

Successful fertilization and fruit set, where the ovary develops into a young fruit, are important for crop yield, as they determine the number and size of fruits and seeds in plants [1]. The development and differentiation of anthers and pistils, the male and female reproductive organs, respectively, are precisely regulated for successful fertilization. In cultivated strawberry (*Fragaria × ananassa*), which is a commercially important soft fruit crop that produces berries rich in beneficial vitamins, nutrients and phenolic compounds, both environmental and genetic factors influence anther development and pollen quality, which are important for a high fruit setting rate [2]. The diploid woodland strawberry *Fragaria vesca* is used as a model plant for gene functional studies and flower and fruit development because it has a small genome, is easily transformed and contains few duplicated regions compared with the genomically more complex octoploid cultivated strawberry [3]. Several transcriptomic analyses of *F. vesca* have provided molecular insight into fertilization and early-stage fruit development [4–6]. Recently, gene editing via CRISPR-Cas9 has been successfully used for functional studies of some of the genes involved in fruit development in both *F. vesca* and *F. × ananassa* [7–10].

The phenylpropanoid pathway produces a diverse group of secondary metabolites, including precursor compounds for flavonoid and lignin biosynthesis. The precursors are then modified by enzymes in the flavonoid or lignin biosynthetic pathways to produce metabolites involved in protection against biotic and abiotic stress, plant reproduction and cell wall structure [11, 12]. The secondary cell wall (SCW) consists of cellulose, hemicellulose and lignin and forms between the primary cell wall and the plasma membrane. It is crucial for mechanical strength and maintaining cell shape and function in specialized cells such as fibres and xylem. The deposition of lignin and the formation of SCWs also improve water conductivity and provide stress tolerance in plants. In anthers, the formation of lignified, cellulosic secondary wall thickenings in cells of the endothecium is required for anther dehiscence, creating pressure for pollen exposure and dispersal. This process is precisely controlled, and disruption in the development of these thickenings prevents anther dehiscence and causes male sterility [13, 14]. The receptacle, which is a modified stem tip in strawberry, is topped with dozens of pistils, each containing an individual carpel with an ovary and an ovule. Successfully fertilized ovules develop seeds and produce the phytohormones auxin and gibberellins, which stimulate the receptacle to enlarge into the fleshy structure known as the berry [5, 15–17]. Auxin also signals the ovary wall (carpel wall) to enlarge and develop into dry achenes, which is probably due to cell wall synthesis and extensive lignification [18]. This positive control of early fruit development by auxin ensures that fruit set only occurs after successful fertilization.

MYB transcription factors bind to specific DNA sequences in the promoter regions of target genes that influence numerous processes, including secondary cell wall biosynthesis, abiotic stress tolerance, resistance to biotrophic and necrotrophic pathogens and flower organ development [19, 20]. Several studies have shown that MYB transcription factors regulate phenylpropanoid metabolism, which is important for fertilization and early fruit development [21, 22]. The R2R3-type MYB26 transcription factor regulates anther dehiscence, which is important for successful fertilization, by inducing secondary thickening through the transcription factors NAC SECONDARY WALL THICKENING 1 (NST1) and NST2 in *Arabidopsis* [23–25]. NST1 and NST2, together with SECONDARY WALL-ASSOCIATED NAC DOMAIN PROTEIN1 (NST3/SND1), have also been shown to be involved in secondary cell wall synthesis in the fibres and xylem of inflorescence stems [26].

The MYB46 transcription factor is a key player in the transcriptional network regulating secondary wall biosynthesis in xylem cells of inflorescence stems in *Arabidopsis*, where it acts redundantly with MYB83 [27, 28]. Both *MYB46* and *MYB83* are directly regulated by several NAC transcription factors, such as VASCULAR RELATED NAC-DOMAIN 6 (VND6) and VND7, NST3/ANAC012/SND1, NST1 and NST2 [27, 29]. MYB46 directly binds and activates transcription factor genes and genes involved in the biosynthesis of cellulose, hemicellulose and lignin [29–31]. The role of MYB46 in secondary cell wall polymerization and the biosynthesis of cellulose and lignin has been demonstrated in several species, including birch and apple, where MYB46 activity also improves salt and osmotic stress tolerance [32, 33]. In *F. vesca*, *FvMYB46* was recently found to be a direct target of the NAC transcription factor FvVND4c and to regulate SCW thickening and flavonoid accumulation when it is overexpressed [34].

Most studies functional studies of the R2R3 MYB transcription factor MYB46 in different plant species have been done using overexpression constructs. In this study, we designed a CRISPR-Cas9 construct with an endogenous FvU6 promoter to efficiently knock out *FvMYB46* to study gene function in its native context in flowers. Transcriptomic analysis using RNA-seq and metabolomic profiling of flower tissue using HPLC were used to identify the downstream pathways regulated by FvMYB46. Our molecular work combined with phenotypic analysis reveal the effect of FvMYB46 activity on biosynthetic pathways, and fertilization and fruit set in *F. vesca.*

## Results

### Expression of *FvMYB46* in different *F. Vesca* tissues

**Fig. 1:**
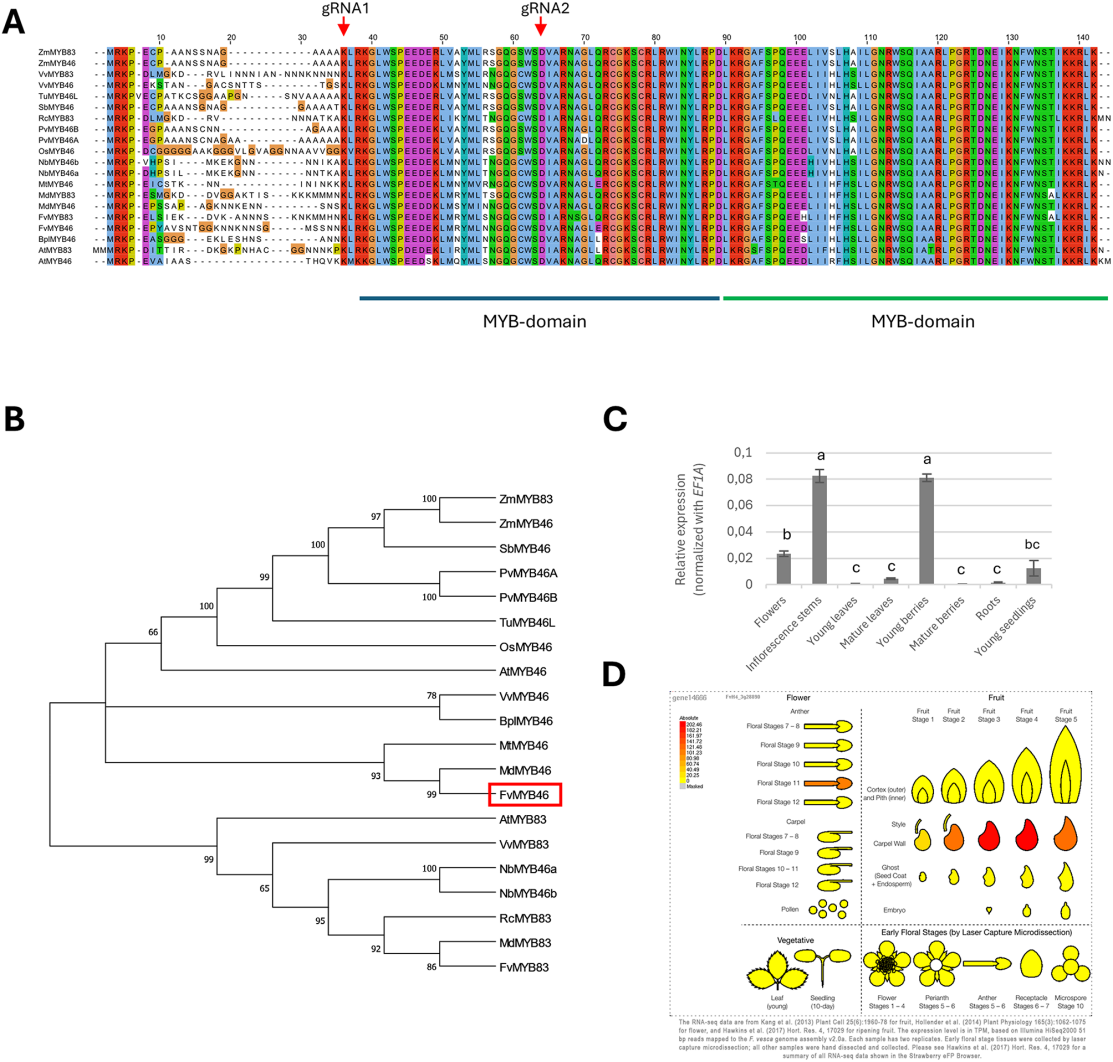
MYB46-homologues from different plant species. (**A**) Alignment of sequences of the N-terminal region comprising MYB domain 1 and MYB domain 2 from functionally analysed MYB46 proteins from different plant species. Arrowheads indicate deletions mediated by gRNA1 and gRNA2. (**B**) Phylogenetic analysis of protein sequences from A) using the neighbor joining method with 1000 bootstrap samples. **C**. qRT‒PCR quantification of *FvMYB46* in different tissues of wild-type *F. vesca*. Expression was normalized with *EF1A*. Different letters above the bars indicate significant difference (*p* < 0.01) based on Tukey pairwise comparisons test. The error bars represent ± standard error of 3 biological replicates. **D**) Expression analysis of *FvMYB46* in different organs and at different stages of flower and fruit development using the eFP browser

To identify *MYB46* homologues in *F. vesca*, we used the *MYB46* nucleotide sequence from *Arabidopsis* (AT5G12870) for BLASTn searches against the *F. vesca* nr genome sequence database at NCBI. This identified the two *F. vesca* transcripts FvH4_3g28890 and FvH4_7g01020 as potential *AtMYB46* homologues. A multiple sequence alignment of the protein sequences of potential *F. vesca* MYB46 homologues with the MYB46 and MYB83 transcription factors (Fig. 1a) with known functions from other plants revealed that FvH4_3g28890 clustered with the MYB46 group and was named FvMYB46, whereas FvH4_7g01020 clustered with the MYB83 group and was named FvMYB83 (Fig. 1b). The expression profile of *FvMYB46* in different *F. vesca* tissues obtained via qRT‒PCR analysis revealed lowest relative expression levels of *FvMYB46* in roots, young leaves and mature berries and slightly greater expression in mature leaves and seedlings. The expression was moderate in open flowers and highest in inflorescence stems and young berries (Fig. 1c). Querying the eFP (electronic Fluorescent Pictograph [35]) browser, revealed that *FvMYB46* has the highest expression in anthers at stage 11 of flower development before fertilization and in carpel walls (ovary walls) during early-stage fruit development after fertilization (Fig. 1d) [5, 36].

### CRISPR-Cas9 knockout of *FvMYB46*

**Fig. 2:**
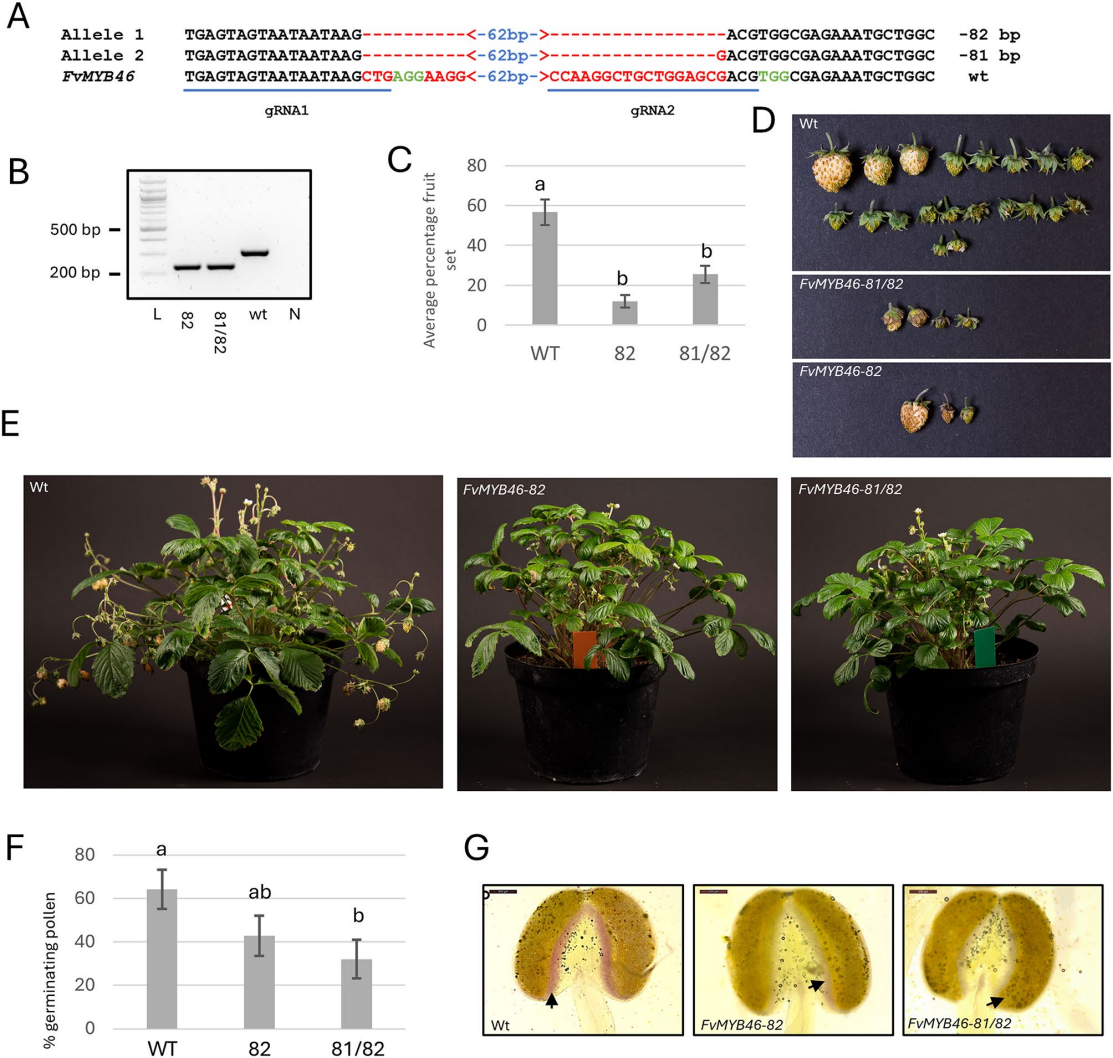
CRISPR Cas9-deletions of *FvMYB46* results in reduced fruit set and anther lignification. (**A**) Sequence alignment of the *FvMB46-81* and *FvMYB46-82* deletions with wt. The two gRNA sequences are underlined. The PAM sequences are colored in green, and the deleted region in red. (**B**) Expression of *FvMYB46* in flowers of WT and mutant plants, analysed by RT-PCR with gene-specific primers flanking the gRNA1 and gRNA2 target sequences. L, 100 bp DNA ladder; 82, *FvMYB46-82* mutant; 81/82, *FvMYB46-81/82* mutant; wt, wild type; N, water control. (**C**) The number of mature berries from the wild type, *FvMYB46-82* and *FvMYB46-81/82* mutants was divided by the total number of flowers to determine the fruit set. The graph shows the average percentage of fruit set per genotype ± standard error, *n* = 12. Different letters above the bars indicate significant difference (*p* < 0.01) based on Tukey pairwise comparisons test. (**D**) Representative image of fruit yield from wt (top), *FvMB46-81/82* (middle) and *FvMYB46-82* (bottom) plants. (**E**) Representative images of wild-type (left) and *FvMYB-82* (middle) and *FvMB46-81/82* (right) plants. (**F**) Frequency of flowers with germinating pollens from wild type and mutants ± Standard error, *n* = 28. Different letters above the bars indicate significant difference (*p* < 0.01) based on Tukey pairwise comparisons test. (**G**) Anthers from wild-type (left), *FvMYB46-82* (middle) and *FvMB46-81/82* (right) plants. The arrows show stained vascular bundles and endothecium facing the connective tissue

We used CRISPR-Cas9 to introduce gene deletions specifically into *FvMYB46* for functional analyses. The AtU6 promoter is frequently used to drive gRNA expression for CRISPR genome editing in dicot plants, but endogenous promoters have been shown to be more efficient in many species [37, 38]. To identify endogenous FvU6 promoters, we used *U6* snRNA genes from *Arabidopsis* and *T. aestivum* in BLAST searches against the *F. vesca* genome. In transient expression analyses, the FvU6-1 promoter resulted in stronger and more robust expression of gRNAs than the AtU6 promoter (Supplementary methods, Figure S1); hence, FvU6-1 was selected for the expression of two gRNAs targeting the first exon of *FvMYB46* in CRISPR-Cas9 constructs used for *Agrobacterium*-mediated stable transformation of *F. vesca*. PCR screening using primers flanking the gRNA target region and direct sequencing of 50 primary transformants (T0) identified three different genotypes containing homozygous deletions of 81 bp (*FvMYB46-81*) and 82 bp (*FvMYB46-82*) or biallelic 81/82 bp (*FvMYB46-81/82*) deletions in both the *FvMYB46* gene and cDNA from flowers (Fig. 2a, b and Figure S2 b, c). The wild-type full-length *FvMYB46* gene encodes a protein of 335 aa, whereas the 81 bp deletion creates an in-frame deletion of 27 aa, resulting in a putative protein of 308 aa. The 27 aa deletion removes most of the first of the two N-terminal MYB/Sant/HTH domains important for DNA-binding and protein‒protein interactions. In contrast to the 81 bp deletion, the 82 bp deletion creates a putative out-of-frame translation product of 50 aa, where only the first 28 aa are identical to those of the wild-type protein (Figure S2 a), thus containing none of the conserved MYB domains. To avoid any influence on downstream molecular and phenotypic analyses, we removed the T-DNA containing the CRISPR-Cas9 cassette by selfing primary transformants of the T0 generation. Hence, in the T1 generation we selected homozygous *FvMYB46-82* and biallelic *FvMYB46-81/82* plants negative for the T-DNA insertion for further studies (Figure S2 c).

The specificity of the gRNA-directed deletions was determined via PCR amplification of the three top-ranking potential off-target sites for gRNA1 and one site for gRNA2 and sequencing of these PCR products. No mutations except for the intended deletion at *FvMYB46* were found. We also analysed the RNA-seq data for potential mutations at any other sites in the genome but did not identify any off-target mutations or other insertion sites for T-DNA.

### Analysing fruit set in *FvMyb46 *plants

The high expression of *FvMYB46* in inflorescence stems, flowers and young unripe berries compared to other organs and development stages, and the specific expression in anthers and ovary walls suggest a role for *FvMYB46* in fertility or early fruit development. By calculating the frequency of flowers that developed into mature fruits, we observed that the fruit set was significantly lower in the homozygous *FvMYB46-82* and biallelic *FvMYB46-81/82* plants than in the wild type (Fig. 2c). Compared to the wild type, a greater number of the flowers in both mutants were seemingly arrested in early receptacle fruit development, after which they died and dried up in contrast to flowers that developed into normal fleshy fruits (Fig. 2d, e). Reduced fruit set can be a consequence of reduced pollination caused by reduced pollen viability, pollen tube elongation and fertilization. We observed a reduced number of flowers with germinating pollen in both mutant genotypes compared to the wild type (Fig. 2f), suggesting that FvMYB46 is involved in pollen development and/or viability. Except for a lower fruit yield and a greater frequency of berries arrested during early fruit development, we did not observe other morphological deviations in the *FvMYB46-82* and *FvMYB46-81/82* plants during vegetative growth (Fig. 2e).

### Microscopy analyses of secondary cell walls in inflorescences and anthers

Microscopy analyses of *FvMYB46-82*,* FvMYB46-81/82* and wild-type anthers stained with phloroglucinol-HCl were carried out to study the lignin content in the secondary cell walls. Phloroglucinol-HCl staining of buds before anthesis and open flowers revealed modest but specific reduced staining of mutant anthers compared with that of wild-type anthers, especially in endothecium cells facing the connective tissue and vascular tissue of the filament (Fig. 2g). However, the total lignin content did not significantly differ between the flowers of the wild type and mutants (Figure S3), suggesting that the observed modest reduction in lignin content in the cell walls of anthers was not affected at the global level.

### Transcriptional profiling of *FvMYB46-81/82* and *FvMYB46-82* flowers

**Fig. 3:**
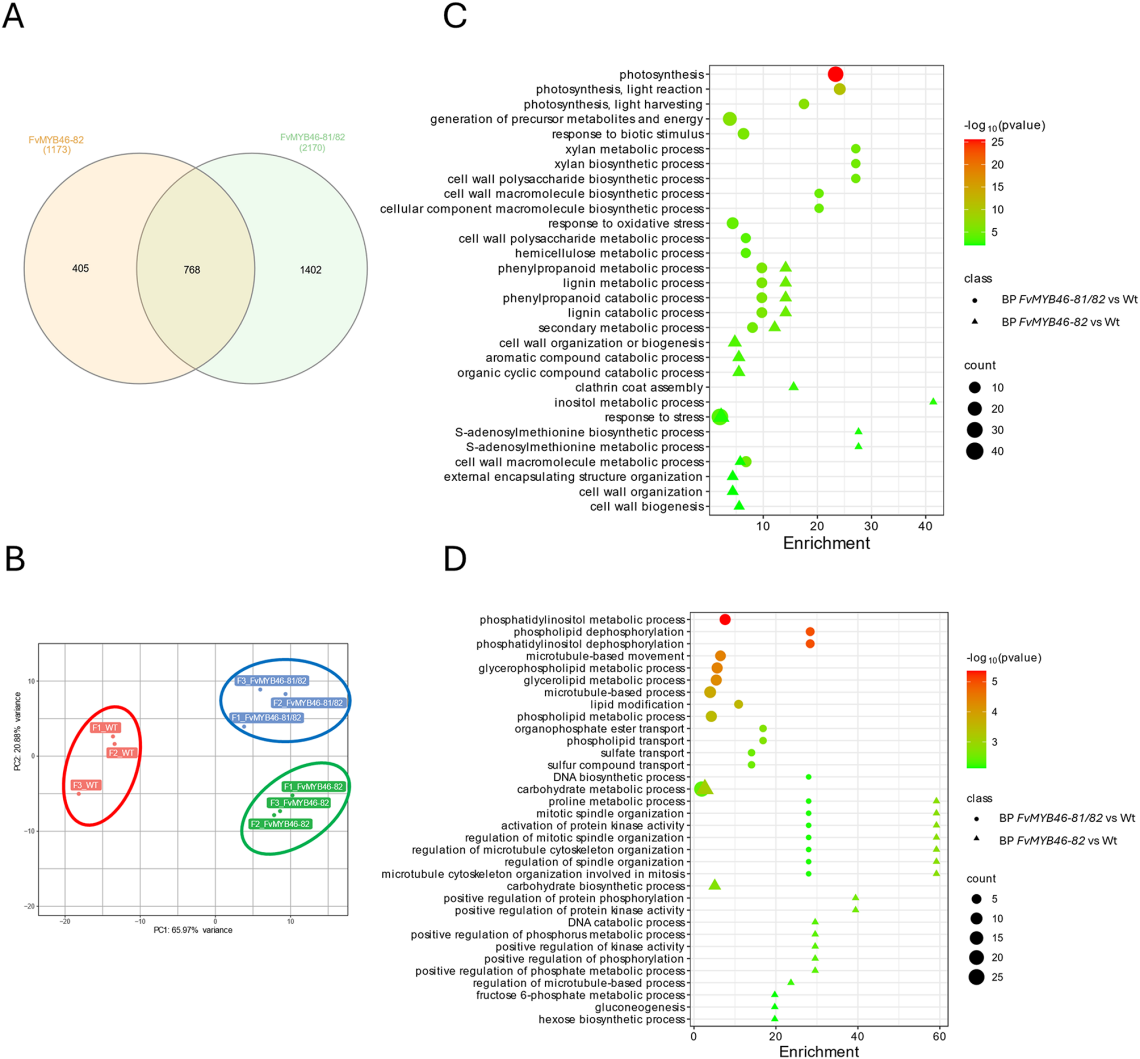
Co-expressed and unique DEGs in flowers of the *FvMYB46-82* and *FvMYB46-81/82* plants. (**A**) Venn diagram of co-expressed and uniquely expressed genes (FDR < 0,01) after pairwise comparison of DEGs. (**B**) Principal component analysis (PCA) of samples based on RNA-Seq data. Pathway enrichment bubble plots comparing enriched GO terms (*P* < 0.01) in the biological process category for *FvMYB86-81/82* vs. wt (circles) and *FvMYB46-82* (with 0.95 confidence ellipses) vs. wt (triangles) for downregulated DEGs (**C**) or upregulated DEGs (**D**). The X-axis shows the fold enrichment values

RNA-seq was then performed on open flowers to identify the genetic pathways affected by *FvMYB46* during early fruit development. A comparison of the differentially expressed genes (DEGs) in the *FvMYB46-82* and *FvMYB46-81/82* plants with those in the wild type (FDR < 0.01) (Supplementary Table 1) revealed that 631 of the annotated transcripts mapped to the *F. vesca* v4.0.a2 transcriptome were downregulated and that 544 were upregulated in the *FvMYB46-82* flowers, whereas 1247 were downregulated and 925 were upregulated in the *FvMYB46-81/82* flowers. A total of 768 genes differentially expressed in the *FvMYB46-82* flowers were also differentially expressed in the FvMYB-81/82 flowers (Fig. 3a; Table 1). Principal component analysis (PCA) separated the wild type and the *FvMYB46-82* and *FvMYB46-81/82* mutants, but formed separate groups, suggesting that differential gene expression was affected by the mutations (Fig. 3b).

**Table 1:** Selected differently expressed genes in flowers of *FvMYB46*-deletion mutants compared to wild type.

Gene ID	Gene Name	Gene bank annotation	log2 FC	log2 FC
			*FvMYB46-81/82*	*FvMYB46-81*
**Signalling and transcription factors**				
FvH4_3g28890	*Fv* *Myb46*	Myb Transcription Factor	-1.156	-0.540
FvH4_7g01020	*Fv* *Myb83*	Myb Transcription Factor	NS	NS
FvH4_3g45450	*Fv* *MYB5*	Transcription Repressor Myb5	NS	-0.143
FvH4_3g34960	*Fv* *MYB59*	Transcription Factor Myb59	NS	0.217
FvH4_5g17111	*MYB3-like*	Transcription Factor Myb3-Like	-0.680	-0.651
FvH4_7g08972		L10-Interacting Myb Domain-Containing Protein-Like	9.278	9.226
FvH4_6g33050	*NAC37*	NAC domain-containing protein 37	6.090	6.094
FvH4_6g25450	*PI5K4*	Phosphatidylinositol 4-phosphate 5-kinase 4-like	1.260	2.039
**Phenylpropanoid pathway**				
FvH4_6g16060	*PAL1*	Phenylalanine Ammonia-Lyase	NS	NS
FvH4_7g19130	*PAL2*	Phenylalanine Ammonia-Lyase	-0.549	-0.862
FvH4_2g05780	*CCOAOMT1*	Caffeoyl-Coa O-Methyltransferase	-0.805	-1.066
FvH4_3g06690	*FvHST*	Shikimate O-Hydroxycinnamoyltransferase-Like	NS	-0.754
FvH4_6g28680	*CCR1*	Cinnamoyl-Coa Reductase 1-Like	-0.375	-0.566
FvH4_6g27940	*4CL1*	4-Coumarate--Coa Ligase 1-Like	-0.85	-1.092
**Flavonoid**,** anthocyanin and lignin biosynthesis**				
FvH4_7g32990	*COMT1*	Caffeic Acid 3-O-Methyltransferase	NS	-0.432
FvH4_7g25890	*CHI3*	Chalcone-Flavanone Isomerase 3	-0.559	-0.823
FvH4_7g20870	*FvCHI1*	Chalcone–Flavonone Isomerase 1	-0.579	-0.786
FvH4_7g33840	*FvUFGT*	Anthocyanidin 3-O-Glucosyltransferase 2	-0.951	-1.180
FvH4_5g01170	*FvANS*	Anthocyanidin Synthase	-0.836	-1.036
FvH4_7g01160	*FvCHS1*	Chalcone Synthase	-0.72	-0.960
FvH4_2g39520	*FvDFR*	Dihydroflavonol 4-Reductase	-0.608	-0.914
FvH4_1g11810	*F3H*	Flavanone-3-Hydroxylase	-0.491	-0.692
FvH4_3g40570	*C4H/CA4H*	Trans-Cinnamate 4-Monooxygenase	-0.459	-0.877
Fvh4_3g13000	*FvF3GT2*	Anthocyanidin 3-O-Glucosyltransferase 7-Like	-2.007	-2.22
FvH4_3g02980	*FvANR*	Anthocyanidin Reductase	NS	-0.725
FvH4_2g39620	*EGS1*	Isoflavone Reductase-Like Protein/Eugenol Synthase	-0.534	-0.773
FvH4_1g29330	*C3H*	p-coumaroyl-shikimate/quinate 3-hydroxylase	-0.472	-0.525
FvH4_6g30610	*CSE*	caffeoyl shikimate esterase	-0.405	-0.597
**Cell wall associated genes**				
FvH4_6g16911	*FvEXPA18*	Expansin-A9-Like	2.452	2.682
FvH4_3g36410	*FvEXPA9*	Expansin A6	-0.666	-0.679
FvH4_6g15450	*IRX9*	Probable Beta-1,4-Xylosyltransferase Irx9	NS	-0.619
FvH4_1g00670	*CESA7/IRX3*	Cellulose Synthase A Catalytic Subunit 7	-0.527	-0.822
FvH4_3g07420	*CESA8/IRX1*	Cellulose Synthase A Catalytic Subunit 8	-0.278	-0.579
FvH4_3g38140	*Lac4/IRX12*	Laccase-4	-0.813	-1.059
FvH4_6g11960	*Lac4/IRX12*	Laccase-4	-0.531	-0.980
FvH4_4g22570	*IRX10*	Probable beta-1,4-xylosyltransferase IRX10	-0.530	-1.015
FvH4_4g19370	*Chl2*	Chitinase-Like Protein 2	-0.827	-1.294
FvH4_6g12782		Vegetative cell wall protein gp1-like	-7.039	NS
FvH4_3g25190	*FvMT127*	glucuronoxylan 4-O-methyltransferase 1-like	3.037	3.4649
FvH4_5g28110	*GXM1*	Glucuronoxylan 4-O-methyltransferase 1	-0.711	-1.0269
FvH4_5g32940	*GXM3*	Glucuronoxylan 4-O-methyltransferase 3	-0.298	-0.4394
FvH4_6g15700	*FvFLA12*	fasciclin-like arabinogalactan protein 12	-0.616	-0.9427
FvH4_2g30720	*FvFLA14*	fasciclin-like arabinogalactan protein 14	-1.088	-0.0452
FvH4_1g16440	*IX15L*	protein IRX15-LIKE-like	-0.640	-0.9233
FvH4_4g33970	*FvPME6*	Pectinesterase	-1.102	-0.8178
FvH4_4g20220	*FvPER40*	Peroxidase 40	-1.626	-1.8997
FvH4_5g15430	*PLY_LILLO*	Pectate lyase	2.040	2.0496
**Carbohydrate metabolism**				
FvH4_2g28820	*SPSA1*	Probable sucrose-phosphate synthase 1	1.862	2.039
FvH4_1g10110	*MSSP2*	Monosaccharide-sensing protein 2-like	3.054	3.191
FvH4_5g05160	*FvSWEET5*	Bidirectional sugar transporter SWEET5	2.285	2.303
FvH4_4g15170	*FvSTP20*	Sugar transporter	2.548	2.875
FvH4_4g15160	*FvSTP18*	Sugar transporter	2.388	2.434
FvH4_4g15172	*FvSTP14*	Sugar transporter	1.264	1.921
FvH4_6g19270	*FvINV1*	Putative Beta-Fructofuranosidase/Cell wall invertase	9.639	8.776
FvH4_6g19271	*FvINV3*	Putative Beta-Fructofuranosidase/Cell wall invertase	6.25	5.567
FvH4_1g28890	*FvFRK3*	Fructokinase-6, chloroplastic	-0.397	-0.532
FvH4_2g00080	*FvFRK5*	Fructokinase-5	-0.941	-1.026
FvH4_6g12281	*FvFLK1*	fructokinase-like 1, chloroplastic	-0.548	-1.140
FvH4_4g18710	*FvSUS2*	Sucrose synthase 2	-7.584	-4.424
FvH4_1g09360	*FvSUS_MEDSA*	Sucrose synthase	-0.296	-0.181
FvH4_1g04190	*FvUGFGT*	UDP glucose: flavonoid 3-O-glucosyltransferase	-0.858	-1.074

*Differently expressed genes with log2 fold change (FC) values and FDR cut-off of 0*,*01. NS: Not statistically different*

### Enrichment analysis of functional terms in DEGs

GO (Gene Ontology) enrichment analysis of the downregulated DEGs (P value < 0.01) in the biological process category for the *FvMYB46-82* flowers revealed that terms related to secondary metabolites, the cell wall, lignin biosynthesis and metabolism, and phenylpropanoid biosynthesis and metabolism were among the most enriched. The most enriched GO terms in the biological process category of the downregulated DEGs for *FvMYB46-81/82* flowers (Fig. 3c, Supplementary Table 2) were similar to those of *FvMYB46-82*, except that the GO terms ‘photosynthesis’, ‘xylan biosynthesis’ and ‘xylan metabolic process’ were also enriched. For the upregulated genes, the terms ‘phosphatidylinositol metabolic process’ and ‘phosphatidylinositol dephosphorylation’ were enriched for the *FvMYB46-81/82* flowers, whereas ‘carbohydrate metabolism’ and terms related to microtubules were enriched in both mutants (Fig. 3d, Supplementary Table 3).

For the molecular function category, the most enriched GO terms for the downregulated DEGs in the flowers of both mutants were related to ‘oxidoreductase activity’. For the cellular component category (Supplementary Table 4), ‘apoplast’ and ‘extracellular region’ were the most enriched categories in both the *FvMYB46-81/82* and *FvMYB46-82* flowers.

**Fig. 4:**
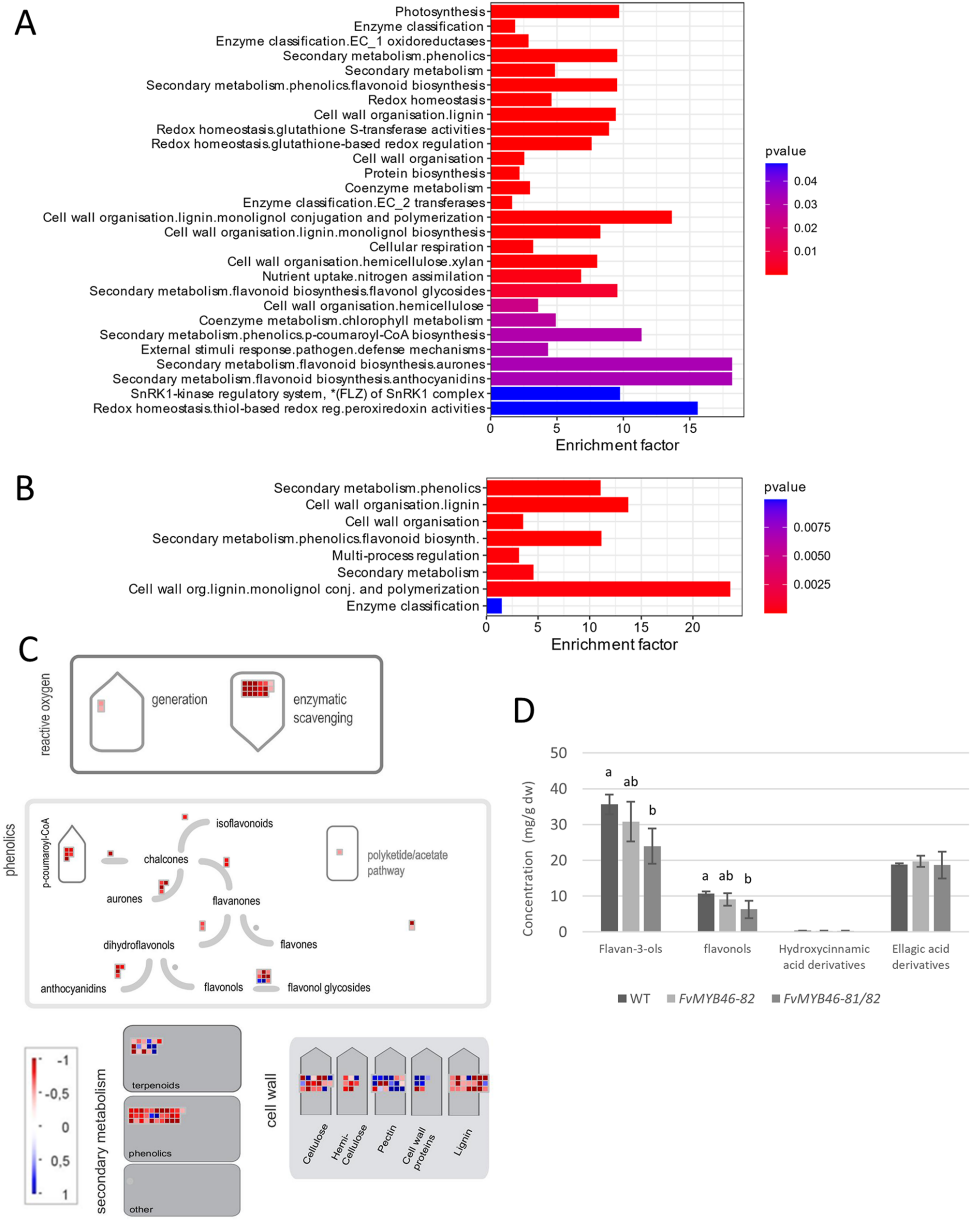
Mercator and MapMan visualization of enriched metabolic pathways in the *FvMYB46* mutants. Mercator enrichment analysis of downregulated DEGs in the *FvMYB46-81/82* (**A**) and *FvMYB46-82* (**B**) mutant plants compared with the wild type. (**C**) Genes differentially expressed in the *FvMYB46-81/82*-mutants displayed onto metabolic pathways using the MAPMAN software: secondary metabolism, including phenolics and terpenoids; the flavonoid pathway; redox homeostasis; and the cell wall. Blue cells: upregulated in *FvMYB46-81/82* compared with the wild type; red cells: downregulated in *FvMYB46-81/82* compared with the wild type (FDR < 0,01). (**D**) Concentrations of flavan 3-ols, flavonols, ellagic acid derivates and hydroxycinnamic acid derivates in flowers determined using HPLC in mg/g dry weight. Different letters above the bars for each compound indicates significant difference (*p* < 0.05) based on Tukey pairwise comparisons test

A more detailed functional categorization of DEGs and associated metabolic pathways was performed with Mercator enrichment and MapMan4 analysis (Fig. 4a-c, Figure S4, S5). This analysis confirmed the significant downregulation of genes involved in cell wall organization, lignin and monolignol conjugation and polymerization in the flowers of both mutants compared with those of the wild type. The results also revealed significant enrichment of genes involved in secondary metabolite metabolism, such as phenolic, flavone and flavonoid biosynthesis, and significant enrichment of genes involved in redox homeostasis, especially glutathione S-transferase activities, among the downregulated genes, while pectin and other cell wall genes were upregulated.

### Analysis of phenolic compounds

HPLC analysis of flower tissue identified 18 phenolic compounds that were grouped into flavonols, flavan-3-ols and derivatives of ellagic and hydroxycinnamic acids. The flavan-3-ols and flavonols were lower in both mutants than in the wild type, although the lowest concentration was detected in the *FvMYB46-81/82* mutant (Fig. 4d). Among the individual compounds, epicatechin and proanthocyanin 3, belonging to the flavon-3-ols group, and quercetin-glucoside and kaempferol-3-coumaroylhexoside, belonging to the flavonol group, were the most downregulated (Figure S6). The concentrations of hydroxycinnamic acid derivatives were relatively low, with minor differences between the genotypes, whereas no significant differences were detected for ellagic acid derivatives.

## Discussion

### FvMYB46 regulates the fruit setting rate

The R2R3 MYB transcription factor MYB46 is a central regulator of lignin, xylan and flavonoid biosynthesis and secondary cell wall formation in xylem vessels and fibres, but little is known about its activity in flowers and berries in *F. vesca*. In this study, we used CRISPR-Cas9 constructs with an endogenous FvU6-1 promoter directing gRNA-expression to introduce in-frame (*FvMYB46-81*) or out-of-frame knockout (*FvMYB46-82)* deletions of *FvMYB46*. The reduced pollen germination and fruit setting with a high frequency of berries arrested at the receptacle stage in *MYB46-81/82* and *MYB46-82* plants suggest that *FvMYB46* regulates fertility and/or early fruit development in *F. vesca*. Before fertilization, *FvMYB46* was expressed specifically in anthers at the flower development stage 11. At this stage, the endothecium cells of the anther increase in size due to the formation of lignified, cellulosic secondary wall thickenings necessary for anther dehiscence and pollen dispersal [13, 36]. In *Arabidopsis*, both repression and overexpression of MYB46 are associated with sterility due to anther indehiscence and ectopic secondary cell wall formation in stamens and carpels, respectively [27, 39]. Recently the overexpression of FvNST1b, the closest homologue to AtNST1 which directly regulates *MYB46* and anther dehiscence and fertility in *Arabidopsis*, was shown to promote secondary cell walls in various tissues, including anthers and ovules in *F. vesca* [40]. *FvMYB46* expression in the carpel walls of green berries coincides with the activation of cell wall synthesis genes and extensive lignification when carpels develop into dry achenes after fertilization [18]. However, we did not observe any effect on the total lignin content in flowers of *FvMYB46* mutants, although we observed a modest reduction in the lignin content in endothecium cells facing the connective tissue of the filament, suggesting that FvMYB46 positively regulates the lignification of secondary cell walls in these tissues. The positive effect on biosynthesis of secondary cell wall components was also reflected on the transcriptomic level. Specifically, phenylpropanoid pathway genes encoding enzymes involved in biosynthesis of the lignin precursors monolignols, as well as peroxidases and laccases which polymerizes monolignol to lignin in the apoplast, were downregulated in the *FvMYB46*-mutants (Table 1; Figs. 3c and 4a-c) [41, 42]. The GO terms ‘xylan biosynthesis’ and ‘xylan metabolic process’ were also enriched among downregulated genes, suggesting a role for FvMYB46 in regulation of hemicellulose which is an important component of secondary cell walls (Table 1).

### *FvMYB46* regulates flavonoid biosynthesis and stress signalling genes in fertilization and early fruit development

GO terms related to secondary metabolite biosynthesis and the flavonoid branch of the phenylpropanoid biosynthesis pathway, as well as genes involved in the enzymatic scavenging of reactive oxygen species, were also enriched among downregulated DEGs in the *FvMYB46*-mutants. In *Fragaria × ananassa*, the general phenylpropanoid pathway and flavonoid biosynthesis genes *chalcone synthase (CHS)*, *Phenylalanine Ammonia- Lyase 2 (PAL2), CHALCONE ISOMERASE (CHI), eugenol synthase 1 (EGS1), Caffeoyl-Coa O-Methyltransferase (CCoAOMT)* and *flavanone-3-Hydroxylase (F3H)* are expressed in flowers and during early fruit development [43, 44]. Consistent with the suggested role of FvMYB46 in regulating phenylpropanoid biosynthesis during early fruit development, these genes were downregulated in the *FvMYB46* mutants. The silencing of chalcone synthase, which is the first step in flavonoid biosynthesis, leads to impaired pollen tube growth in tomato [45] and reduced levels of anthocyanins, flavonols, and proanthocyanidins in *Fragaria × ananassa* [46]. Metabolite profiling confirmed that the levels of the flavonols quercetin-glucoside and kaempferol-3-coumaroylhexoside, and the flavan-3-ols proanthocyanidins 3 and epicatechin were reduced in open flowers of the *FvMYB46* mutants. These findings suggest that FvMYB46 regulates flavonoid biosynthesis during fertilization and early fruit development, most likely in the anthers and carpel walls, thus likely affecting the observed fruit set phenotype. Flavonoids are important for stress tolerance, pollen development, pollen viability, pollen germination and pollen tube growth and thus play important roles in plant reproduction [47]. For example, in tomato, the reduced flavonol content in the pollen and pollen tubes of the *anthocyanin reduced (are)* mutant lacking a functional F3H was associated with reduced number of viable pollen grains, slower pollen tube growth, reduced pollen germination rate and a greater abundance of total ROS and H_2_O_2_ under normal and heat stress conditions. This is consistent with flavonols controlling pollen development and pollen tube growth by scavenging reactive oxygen species (ROS) [48]. Compared with the wild type, the *are* mutant also had smaller fruits and a lower seed set.

Several studies have shown that MYB46 enhances stress tolerance by e.g. inducing the expression of genes encoding ROS-scavenging enzymes [32, 33, 49]. Interestingly, glutathione-S-transferases that are detoxifying and ROS-scavenging enzymes were enriched among the downregulated genes in the *FvMYB46* mutants and were coexpressed with *FvMYB46* in the carpel wall/ovary wall of the developing embryo. Thus, reduced stress resistance or impaired ROS signalling may result in the reduced fruit set phenotype observed in the *FvMYB46*-mutants. Several other genes important for successful fertilization were differentially expressed in the *FvMYB46*-mutants. For example, expansins were differentially regulated in the *FvMYB46* flowers. Expansins are believed to be involved in softening the cell walls of the stigma, facilitating the growth and penetration of the pollen tube and successful fertilization [50, 51]. Pollen development and anther dehiscence are tightly controlled, and both premature and delayed processes can cause sterility. FvMYB46 activity might be important for this control, as we observed significant reduction in the fruit set and pollen germination associated with modest downregulation of flavonoid, lignin and secondary cell wall associated genes, and reduction of flavonoid and lignin content in flowers and endothecium layer respectively.

In both *FvMYB46* deletion mutants genes encoding enzymes for sugar metabolism like fructokinases (FRKs) and sucrose synthases (SUS) were among the downregulated genes. Sucrose is transported from leaves to flowers and fruits, and then hydrolysed to fructose and glucose in the extracellular matrix by cell wall invertases (CWINs), or to fructose and uridine 5ʹ-diphosphate-d-glucose (UDPG) used for respiration and cellulose and hemicellulose biosynthesis, respectively by SUSs in the cytoplasm. The fructokinases phosphorylate fructose to fructose-6-phosphate (F6P) which is required for energy production and feeding downstream pathways including generation of secondary metabolites and cell wall components through the phenylpropanoid pathway [52, 53]. Sucrose signalling, where sucrose or variations in the sucrose/hexose ratio induce transcription factor expression, have been shown to regulate phenylpropanoid metabolism and flavonoid biosynthesis in several plant species [54–56]. In potato, sucrose stimulate expression of the MYB transcription factor Anthocyanin 1 (StAN1) which together with StbHLH and StWD40 activate phenylpropanoid pathway genes, but also induce expression of invertase and sucrose synthase genes [57]. The reduced expression of 2 of 5 annotated SUSs and 3 of 5 FRKs in flowers of the *FvMYB46* mutant, suggest a similar mode of action in *F. vesca*, where FvMYB46 regulate both phenylpropanoid biosynthetic genes and genes involved in carbohydrate metabolism. SUS activity have also been shown to increase cellulose biosynthesis and secondary cell wall formation in developing xylem vessels and fibres [58, 59]. In tomato, inhibition of a fruit specific SUS caused reduced sucrose import capacity of young fruit from the phloem and reduced fruit set [60]. Several studies have shown that FRKs are important for efficient fruit set by facilitating unidirectional flux from sugar metabolism in the developing fruit to downstream biosynthesis of cell wall components and energy [52, 53]. In summary, mutations in both FRKs and SUSs causes similar fruit set phenotypes as observed for our *FvMYB46*-mutants and causes similar secondary cell wall phenotypes in phloem and xylem tissues as reported for MYB46 mutants in several species. The downregulation of SUSs and FRKs in the *FvMYB46*-mutants therefore suggest that FvMYB46, at least partly, regulates the cross talk between carbohydrate metabolism and phenylpropanoid biosynthesis in early fruit development in *F. vesca*.

The expression of the CWIN homologues *FvINV1* and *FvINV3* and the sugar transporters *FvSTP14*, *FvSTP18* and *FvSTP20* were strongly upregulated in the flowers of *FvMYB46*-mutants, suggesting that FvMYB46 has a negative regulatory effect on expression. In *Fragaria × ananassa*, CWIN and sucrose transporter expression increase, while SUS expression decrease through fruit development and ripening [61]. This is consistent with the reduced expression of SUSs and FRKs and increased expression of *FvINV1* and *FvINV3* in flowers of *FvMYB46* mutants, thereby causing premature repression and activation of these genes respectively. The expression analysis of wild type tissues in our study shows *FvMYB46* expression peaks in unripe berries, before it is shut down in ripe berries. CWINs are involved in biotic and abiotic stress responses and are the main driver of sucrose uptake in strawberry fruit by hydrolysing sucrose to fructose and glucose in the apoplastic space, generating a sucrose gradient that drives sucrose uptake [62]. Thus, we suggest that FvMYB46 positively regulates SUS and cell wall biosynthesis, FRK and phenylpropanoid expression for efficient fertilization and fruit set early in development, while enabling increased sucrose uptake and sugar signalling by relieving repression on CWINs in ripe berries.

### Redundancy and dominant negative effects

The modest effect on gene expression and the lignin and flavonoid levels in the *FvMYB46* deletion lines is consistent with the small increase in the total lignin levels in both *Arabidopsis* and *F*. *vesca* resulting from MYB46 overexpression observed in previous studies [30, 34]. This might be due to redundancy with the endogenous paralogue *FvMYB83* as observed in *Arabidopsis* [27, 28], which is coexpressed with *FvMYB46* in flowers and during early fruit development in this study. However, the observed fruit-setting phenotype and significant effects on gene expression of biosynthetic pathways suggest that FvMYB83 or other potential homologues is not completely redundant with FvMYB46 in flowers, possibly because the expression of *FvMYB83* is lower than that of *FvMYB46*. Interestingly, we observed a stronger effect on pollen germination and a greater number of DEGs with a stronger up- or downregulation in flowers of biallelic *FvMYB46-81/82* than in *FvMYB46-82*. We hypothesize that the expressed in-frame gene product *FvMYB46-81* with a deletion in the conserved first MYB DNA-binding domain and intact second domain can compete with FvMYB83 by binding to native target promoters or by interacting with other proteins, despite being nonfunctional as transcription factor. A similar effect was observed for the ectopically expressed MYB46 protein fused to a repression domain in Arabidopsis [27]. At the transcriptomic level, there were a significant number of overlapping DEGs between the mutants, and the majority of GO terms were similar for both mutants, although some were unique, e.g. in the *FvMYB-81/82*-bp mutant, GO terms such as ‘photosynthesis’ was enriched in the downregulated DEGs and terms related to ‘phosphatidylinositol’ was enriched in the upregulated DEGs. However, although these DEGs and the stronger up- or downregulation of similar DEGs in *FvMYB46-81/82* flowers likely explain the slightly different effects on the production of phenolic compounds and pollen germination between the mutants, they are not essential for the major fruit set phenotype observed in both mutants.

## Conclusions

In summary, our functional analysis of CRISPR-Cas9-mediated deletion mutants have demonstrated that FvMYB46 regulates pollen germination and fruit set in *F. vesca*. Transcriptional profiling of mutant flowers revealed that FvMYB46 positively regulates genes involved in secondary cell wall formation, pollen tube growth, scavenging of reactive oxygen species and the phenylpropanoid pathway, including lignin and flavonoid biosynthesis. The role of FvMYB46 in flavonoid biosynthesis was supported by metabolite profiling, which demonstrated a reduction of flavonols and flavan-3-ols in mutant flowers. Genes associated with carbohydrate metabolism and sugar signalling were generally upregulated in *FvMYB46*-mutants, although enzymes previously reported to affect fruit set like sucrose synthase and fructokinases were downregulated. Together, these results suggest that FvMYB46 controls fertility and efficient fruit set by regulating the crosstalk between carbohydrate metabolism and signalling, and secondary cell wall biosynthesis and flavonoid biosynthesis, in flowers and early fruit development in *F. vesca*.

## Methods

### Construction of CRISPR/ *FvMYB46* knockout plasmids

For stable transformation experiments for the knockout of  *FvMYB46*, we synthesized a construct containing the two individual expression cassettes *FvU6-1*-gRNA1-scaffold-terminator and *FvU6-1*-gRNA2-scaffold-terminator (Supplementary methods), with GenArt (Thermo Fisher). The fragment was then cloned into the pCAS9-TPC vector [63] using PacI, resulting in the pCAS9-TPC/*MYB46*_2XgRNA construct, and transformed into *Agrobacterium tumefaciens* strain GV3101 for transformation of *F. vesca* Hawaii-4. The sgRNA1 (TGAGTAGTAATAATAAGCTG) and sgRNA2 (CCAAGGCTGCTGGAGCGACG) sequences targeting the first exon of *FvMYB46* were designed using the CRISPR-P 2.0 program [64]. CRISPR-P 2.0 was also used to predict potential off-targets for gRNA1 and gRNA2, which were amplified using specific primers (Supplementary Table 5) and sequenced.

### Agrobacterium-mediated stable transformation

Seeds from *F. vesca* Hawaii-4 (accession PI551572) were first germinated on ½ MS (Murashige & Skoog; Duchefa M0222) medium for 30 days. *A. tumefaciens* strain GV3101 containing the pCAS9-TPC/*MYB46*_2XgRNA construct was pelleted and resuspended in co-cultivation medium (MS medium supplemented with 2% sucrose pH 5.8 and freshly added 100 µM acetosyringone) and used for transformation of ~ 1 cm^2^ leaf discs cut from petioles and young leaves. The leaf discs were incubated in *Agrobacterium*-suspension for 60 min at room temperature, dried with paper towels and moved to MS callus inducing medium (MS-medium containing 2% sucrose (pH 5.8), 0.7% agar, 3 mg/L BAP, and 0.2 mg/L IBA). Putative transformants were selected on MS callus inducing media containing 250 mg/L Cefotaxime and 3 mg/L BASTA. After 10 weeks, calluses were transferred to shoot-inducing medium (MS medium supplemented with 2% sucrose (pH 5.8), 0.7% agar, 1 mg/L BAP, 0.2 mg/L IBA, 250 mg/L Cefotaxime and 3 mg/L BASTA). Developed shoots were subsequently transferred to MS root inducing medium (MS medium supplemented with 1% sucrose, pH 5.8, 0.7% agar). Plants approximately 5 cm in height were moved to soil and maintained in the growth room.

### Plant growth and phenotyping

*F. vesca* Hawaii-4 (accession PI551572) plants were grown in topsoil in 400 mL pots cultivated in a growth room with 14 h light (~ 100 mmol m^−2^ s^−1^ photosynthetically active radiation (PAR) at 24 °C) and 10 h darkness (19 °C) at 40–45% relative humidity for phenotypic inspection and for the production of material for all transcriptomic analyses. For runner and seed production, plants were propagated in the greenhouse under standard conditions. Fruit set was determined by calculating the percentage of fruits fully developing to the ripe stage divided by the total number of open flowers. To ensure that all flowers were at the same developmental stage, they were marked at the pre-anthesis stage. Flowers from 12 wildtype, *FvMYB46-81/82* and *FvMYB46-82* plants were counted over 40 days, starting from day one. All the flowers bloomed during this period, and flowers that resulted in aborted berries and flowers resulting in berries were quantified for 40 days. For pollen tube germination, we used culture medium for in vitro germination of strawberry pollen developed by Yamaguchi et al. (2024) [65] without agar. Briefly, flowers were collected from the greenhouse in the morning, and the pollen was cultured into 10% sucrose and 0.1% boric acid medium. A total of 300 µl of medium was dispensed onto a microscope slide glass cavity (with a 14 mm diameter and 6.5 mm − ^1^ curve surface), and the pollen was brushed into the medium. From each genotype, 28 flowers were investigated in the study. The germination of the pollen was assessed after incubation for 3 h at 22 °C by counting 100 pollen grains from each slide using an optical microscope. The germination of the pollen was evaluated by measuring the diameter and length of the pollen. Pollen with a greater diameter than the pollen grains was considered germinated. The rate of pollen germination was calculated as described [65], the variance between the mutant genotypes and the wild type was tested using one-way ANOVA, and a significance test was performed using Tukey’s multiple-range test (*P* < 0.01 or 0.05) in R software.

### DNA and RNA isolation

Approximately 100 mg of plant tissue was ground in liquid N_2_ and used for DNA extraction using the DNeasy Plant Mini Kit (Qiagen) according to the manufacturer’s instructions. Total RNA was isolated from 100 mg of tissue using the Spectrum Plant Total RNA Kit with minor modifications [66]. Briefly, preheated lysis buffer containing CTAB (2%), PVPP (2%), Tris-HCl (pH 8.0, 100 mM), EDTA (pH 8.0, 25 mM), NaCl (1 M) and β-mercaptoethanol (1%) was mixed with 100 mg of tissue powder, and incubated at 65 °C for 8 min with vortexing for the first 60 s. After centrifugation for 10 min at 13,000 rpm, the supernatant was mixed with an equal volume of chloroform:isoamyl alcohol (24:1) and centrifuged again for 10 min at 4 °C. The supernatant was transferred to the kit’s filtration column (blue retainer ring), and from this step, we followed the manufacturer’s instructions. On-column DNase I treatment was done to ensure DNA-free total RNA.

### Quantitative real-time PCR

A total of 500 ng of RNA from different tissues was used to synthesize cDNA with the iScript cDNA Synthesis Kit (Bio-Rad). Quantitative real-time PCR (qRT-PCR) was performed using the CFX96TM Real-Time System (Bio-Rad) and the SsoAdvanced Universal SYBR Green Supermix (Bio-Rad) with the gene-specific primers in Supplementary Table 5. The relative expression levels were determined using the comparative threshold cycle (∆Cq) method [67]. Elongation factor 1 Alpha (*FvEF1A*) was used as an endogenous control gene to normalize the data [67].

### Transcriptomic profiling using RNA sequencing

Transcriptomic profiling of *F. vesca* Hawaii-4 fully open flowers (0 DPA) containing all flower organs was performed by BGI Genomics Co., Ltd. Five flowers from 5 different wild-type and mutant plants were harvested together for each replicate. Three biological replicates each of wild-type, *FvMYB46-81/82* and *FvMYB46-82* flowers were used for RNA isolation. The RNA samples were sent to the Beijing Genomics Institute (BGI, https://www.bgi.com), Hong Kong, for cDNA library construction, paired-end sequencing (PE100, 40 M) using a DNA nanoball sequencing (DNBSEQ) technology. To produce clean and highly reliable data, SOAPnuke (Version: SOAPnuke.2.2.1 Parameters: -l 15 -q 0.5 -n 0.05 -i (https://github.com/BGI-flexlab/SOAPnuke)) was used to exclude low-quality readings, readings polluted by adapters, and readings with an excessive number of unknown bases.

The sequences were trimmed and quality checked using Trimmomatic (version 0.39) [68] with settings recommended for paired-end reads. An average of 21 million read pairs for each sample were left after filtering, and these were mapped against v4.0.a2 of the *F.* vesca genome [69] using the STAR aligner (version 2.7.11b) [70]. Additional functional annotations of genes were also downloaded for this version of the genome (https://www.rosaceae.org/species/fragaria_vesca/genome_v4.0.a2).

To identify differentially expressed genes (DEGs), the R-wrapper SARTools (version 1.8.1) (ref - 10.1371/journal.pone.0157022) was used with the recommended settings (https://github.com/PF2-pasteur-fr/SARTools/blob/master/template_script_edgeR.r) to run edgeR (version 3.42.4) (ref - doi:10.1093/bioinformatics/btp616). DEGs were scored as significant when the false discovery rate (alpha) was less than 0.05.

### Functional categorization of DEGs using GO and MapMan enrichment analyses

Enriched GO terms were identified using the R package GOstats [71] (version 2.66.0), and FDR cut-off of 0.01 using functional annotation of the *F. vesca* v4.0.a2 transcriptome (www.rosaceae.org). GO pathway enrichment plots were generated with SRplot (www.bioinformatics.com.cn/en). The Mercator4 v6.0 online tool (https://www.plabipd.de/mercator_main.html) was used to functionally annotate and classify all the *F. vesca* transcripts into hierarchically structured bins, combined with DEG analysis and displayed onto metabolic pathways with MAPMAN software version 3.7.0 [72, 73]. DEGs with FDR < = 0.01 were plotted in Venn diagrams using InteractiVenn [74].

### Extraction of phenolic compounds and HPLC

Phenolic compounds were extracted from the samples as described by [75]. A 5 mg aliquot of each sample was subjected to extraction using methanol (HPLC - gradient grade, VWR International LLC, Randor, USA). The extraction process involved homogenization with 10 ceramic balls at a velocity of 6.5 m/s for 30 s, utilizing a VWR Bead Mill MAX homogenizer (VWR International, LLC Radnor, PA, USA). Post-homogenization, samples were iced for 15-minutes duration before they were centrifuged at 15,000 rpm for 3 min, facilitated by an Eppendorf 5417 C centrifuge. The supernatant was decanted into a 5 ml tube, and the remaining residue was redissolved in 400 µl of methanol, followed by homogenization and centrifugation identical to the previous steps. This extraction process was repeated twice, pooling the supernatants each time. The pooled supernatants were subsequently subjected to evaporation in a vacuum centrifuge (Eppendorf concentrator plus) before the dried extracts were reconstituted in 500 µl of a 1:1 (v/v) mixture of methanol and water, assisted by an ultrasonic cleaner (mod. no. USC200TH; VWR International LLC, Randor, USA).

HPLC analysis was performed according to the methodology of [76] using an Agilent 1200 series HPLC system equipped with a binary pump and a diode array detector (DAD) system (Agilent Series 1200, Agilent Technologies, Waldbronn, Germany). Separation was conducted on a Thermo Scientific 50 × 4.6 mm ODS Hypersil column with a particle size of 3 μm (Thermo Fisher Scientific Inc., Waltham, USA) maintained at 25 °C. The mobile phases consisted of 1.5% tetrahydrofuran and 0.25% acetic acid in ultrapure water (mobile phase A) and 100% MeOH (mobile phase B). The gradient for mobile phase A was as follows: 0–1.5 min, 100% A; 1.5–3 min, 100–85% A; 3–6 min, 85–70% A; 6–12 min, 70–50% A; 12–20 min, 50% A; and 20–22 min, 50–0% A. The flow rate was maintained at 2 ml min^−1^, and the injection volume was 20 µl. Compound identification was achieved by analysis of the retention times and UV spectra. The absorbances at 270, 320 and 360 nm were used to calculate concentrations by comparison with commercial standards.

### Gene IDs and accession numbers

The nucleotide sequence of the *Fragaria vesca* transcription factor *FvMYB46* has the gene ID FvH4_3g28890 and the GenBank accession number XM_004291742.2.

## Electronic supplementary material

Below is the link to the electronic supplementary material.

Supplementary Material 1

Supplementary Material 2

Supplementary Material 3

Supplementary Material 4

## Data Availability

The sequencing data supporting the findings of this study were deposited to NCBI’s Sequence Read Archive (SRA) with accession number PRJNA1202907.

## Abbreviations

MYB: v-myb avian myeloblastosis viral oncogene homolog
CRISPR: clustered regularly interspaced short palindromic repeats
gRNA: guide RNA
bp: base pair
ROS: reactive oxygen species
SCW: secondary cell wall
SND1: SECONDARY WALL-ASSOCIATED NAC DOMAIN PROTEIN1
NST1: NAC SECONDARY WALL THICKENING PROMOTING FACTOR 1
TF: transcription factor
VND: VASCULAR-RELATED NAC-DOMAIN
HPLC: High-performance liquid chromatography
BLAST: Basic Local Alignment Search Tool
NCBI: National Center for Biotechnology Information
qRT‒PCR: Quantitative Reverse Transcription PCR
eFP: electronic Fluorescent Pictograph
snRNA: small nuclear RNA
aa: amino acids
T-DNA: transfer DNA
DEGs: differentially expressed genes
FDR: false discovery rate
PCA: Principal component analysis
GO: Gene Ontology
FRK: fructokinase
SUS: sucrose synthase
CWIN: cell wall invertase
UDPG: uridine 5ʹ-diphosphate-d-glucose
F6P: fructose-6-phosphate
HLH: helix loop helix
MS: Murashige & Skoog
BAP: 6-benzylaminopurine
IBA: Indolyl-3-butyric acid
PAR: photosynthetically active radiation
CTAB: cetyl trimethyl ammonium bromide
PVPP: polyvinylpolypyrrolidone
EDTA: ethylenediaminetetraacetic acid
DPA: days post anthesis
DAD: diode array detector
