# Supplementary material for: CRISPR-Cas9-mediated deletions of FvMYB46 in Fragaria vesca reveal its role in regulation of fruit set and phenylpropanoid biosynthesis
Source: BMC Plant Biol. 2025 Feb 25;25:256. doi: 10.1186/s12870-024-06041-0 (PMC11853751; doi:10.1186/s12870-024-06041-0)
Supplement: Supplementary file 1 — Supplementary Material 1 [file 12870_2024_6041_MOESM1_ESM.pdf]

Fig. 2 b

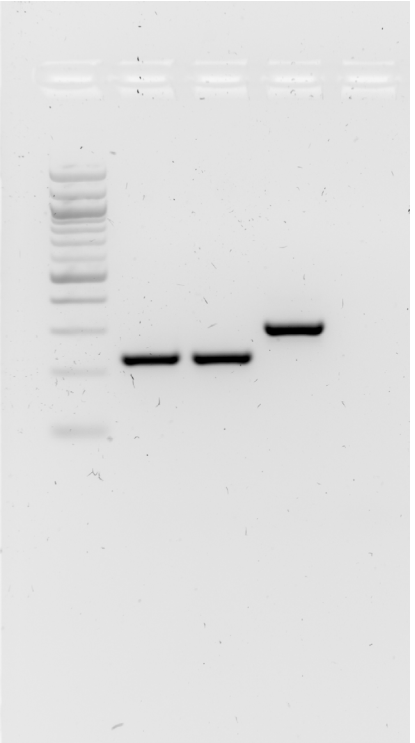

Fig. S2 b

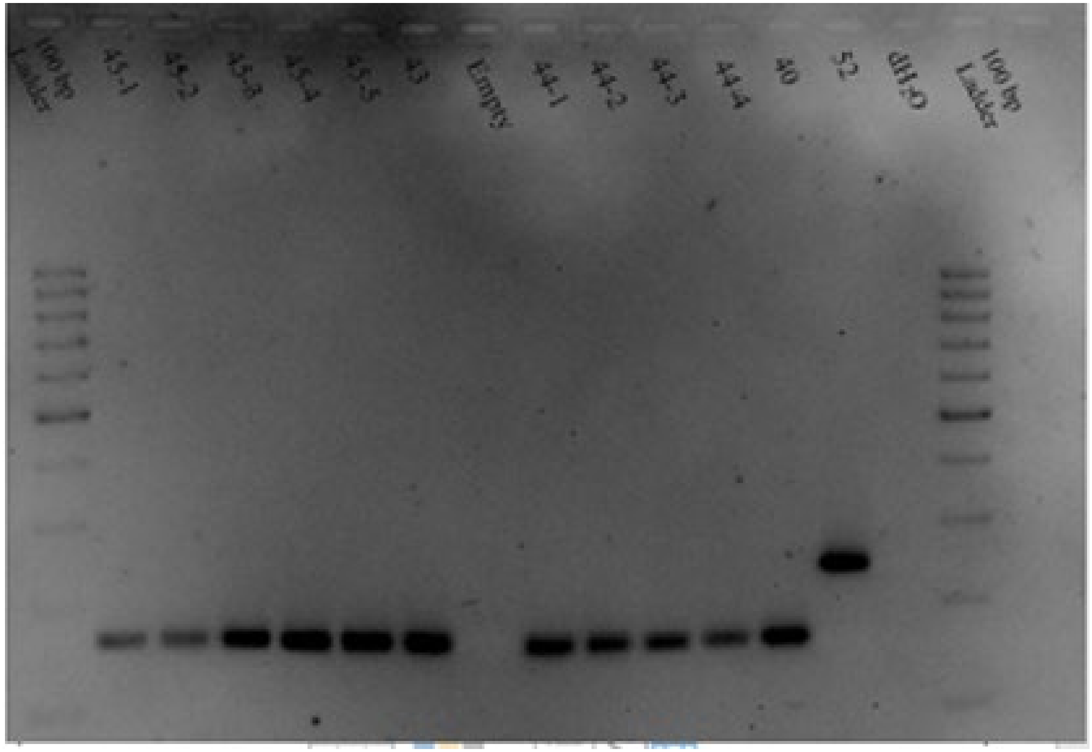

Fig. S2 c (left)

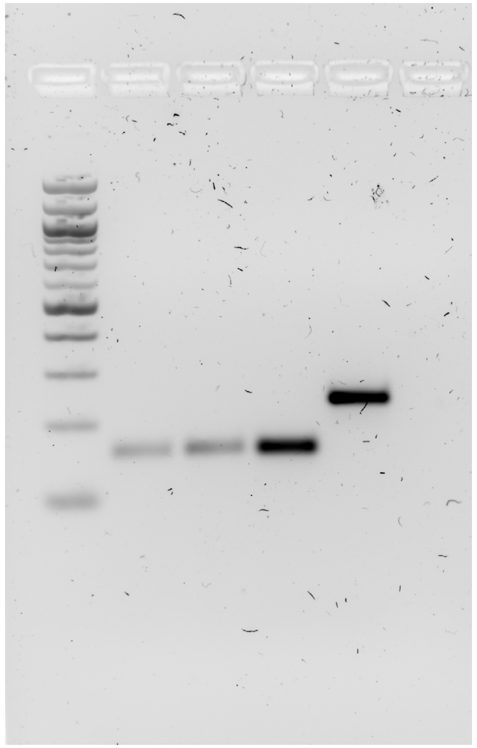

Fig. S2 c (right)

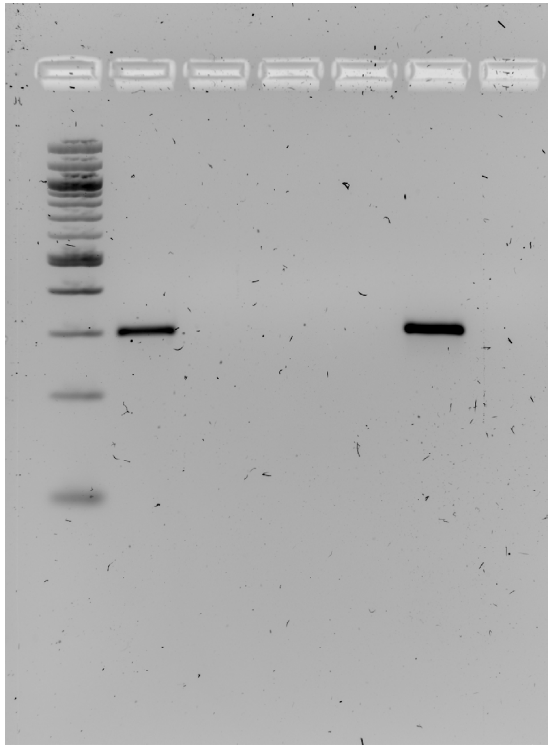

Non-cropped versions of gel images in the manuscript. Figure numbers are identical to the numbers in the manuscript. 2 B) Expression of *FvMYB46* in flowers of WT and mutant plants, analysed by RT-PCR with gene-specific primers flanking the gRNA1 and gRNA2 target sequences. From left to right: 100 bp DNA ladder; *FvMYB46-82* mutant; *FvMYB46-81/82* mutant; wild type; neg control. S2 B) PCR-screening of DNA from T0-plants 40, 43 and 52 (neg) and progeny of 44 and 45-plants with gene specific primers flanking gRNA1 and gRNA2. S2 C) Genotyping of DNA from (left to right) T0 (44), *FvMYB46-81/82, FvMYB46-82* and WT-plants (left). Genotyping of (left to right) T0*, FvMYB46-81/82, FvMYB46-82,* WT and Cas9-plasmid control with Cas9-specific primers.
