# Supplementary material for: CRISPR-Cas9-mediated deletions of FvMYB46 in Fragaria vesca reveal its role in regulation of fruit set and phenylpropanoid biosynthesis
Source: BMC Plant Biol. 2025 Feb 25;25:256. doi: 10.1186/s12870-024-06041-0 (PMC11853751; doi:10.1186/s12870-024-06041-0)
Supplement: Supplementary file 2 — Supplementary Material 2 [file 12870_2024_6041_MOESM2_ESM.pdf]

Supporting methods

Identification and testing F. vesca *U6-promoter sequences for gRNA expression*

Using the *AtU6-26*-gene (X52528), the *AtU6-1* gene (X52527) from *Arabidopsis*, and the *T. aestivum U6* snRNA gene (X63066) in BLAST searches against the nr *Fragaria vesca* nucleotide database at NCBI or the Blast service at the Genome Database Rosaceae (GDR) against the *F. vesca* Whole Genome v4.0.a1 Assembly & Annotation, identified 8 unique *U6* snRNA genes with promoters containing TATA boxes and USE (Upstream sequence elements) known to be important for expression (Supplementary figure 1). ~350 bp upstream sequences from the annotated TSS of these sequences comprising the putative U6-promoters were aligned with MEGA-6. The 329 bp upstream sequence of U6-1, 335 bp of the U6-2 upstream sequence and 335 bp of the U6-3 (acc nr) upstream sequence were selected for transient expression analysis in *F. vesca* berries to identify active promoters. The promoter sequences containing FvU6-BbsI-BbsI-gRNA-scaffold flanked by PacI, attR1 and EcoRI sequences were synthesized at GenArt (Thermo Fisher). The gRNA oligos for *MET1* and *RPPL1*, were annealed to create 20 bp double stranded gRNAs with BsaI overhangs, and subsequently ligated into the BsaI cassette of the pMK-RQ-gRNA expression vectors linearized with BsaI. The U6-promoter-gRNA expression cassettes were then subcloned into the EcoRI site of pFGC - pcoCas9 vector for transient expression experiments.

Transient expression analysis

The constructs were transiently expressed in ripe *F. vesca* fruits by Agroinfiltration. qPCR on cDNA from RNA isolated from fruits 6 days after infiltration using gRNA specific and scaffold specific primers, showed that the FvU6-1 promoter showed the highest and most stable expression compared to the other *F. vesca* promoters and the AtU6-26 promoter (Figure S1 B) suggesting this promoter would be the best option for stable expression of gRNAs in *F. vesca*.

Screening putative CRISPR-Cas9-mutations and outcrossing of T-DNA

Fragaria vesca H4 leaf discs were transformed with pCAS9-TPC-FvU6-1-*MYB46*-2XgRNA using *Agrobacterium* mediated transformation. Developing calluses were propagated on MS-media containing 3 mg/L BASTA, and after shoots appeared, lea*ves* were collected for PCR-screening using primers flanking the gRNA target regions to identify plants with deletions in *FvMYB46*. PCR screening identified 50 T0-plants with a deletion in the expected region. Of these, three (ID #43, #44, and #45) were selfed, and T1 progeny plants were genotyped for deletions in *FvMYB46* and presence of *CAS9* using FvMYB46seqF and FvMYB46seqR2 and *CAS9*-primers (Cas9-F3n and Cas9-R3n) respectively (Figure S2 B, C). The PCRs gave a product of ~160bp suggesting that these plants carried the same deletions in *FvMYB46* as their respective parental line. 12 of the 23 T1 plants with a deletion in *FvMYB46* were negative for *CAS9*, suggesting successful outcrossing of the T-DNA (Figure S2 C).

Supplementary table 5. Oligos used in this study

Primer	Sequence (5´- 3´)
Cas9-F3n	CAACAACTACCACCACGCTCA
LB_Nested_2_R	GAGAGGCGGTTTGCGTATTG
Cas9RTF	TGGTTTCGATTCTCCTACCG
Cas9-R3n	ATCCCTTCCCTTATCCCACAC
HindIII_flank_F_2	GTCCGATTGGAAGCAAGAAC
Flank_upstream_T-DNA-2_F	AATGGTCAAAATACCACATAGGC
gRNA1_MYB46_OT1_F	CGTGTTCTTCCATCCTCACC
gRNA1_MYB46_OT1_R	ACACCGTCAGACTTCAATGG
gRNA1_MYB46_OT2_F	AACAACGGGGAAATAGAAGAGAG
gRNA1_MYB46_OT2_R	TGTAATGCAGGCACTTCCAC
gRNA1_MYB46_OT3_F	TTGCATGCATGTTGATGTTG
gRNA1_MYB46_OT3_R	TTCATGGTTCGCATTCACAG
gRNA2_MYB46_OT1_F	CTCCGGAAACCCAATAAGTG
gRNA2_MYB46_OT1_R	ACGAGGGTTTGTGTTTCGAG
FvMYB46seqF	GAACCCTATGCTGTAAGTAATACCG
FvMYB46seqR2	ACCTCTCTTAAGGTCAGGTCTCAA
TPC_F	TCTTGAATTGGTTTGTTTCTTCAC
TPC_R	TAGACAAGCGTGTCGTGCTC
FvEF1ARTF	GCCCATGGTTGTTGAAACTTT
FvEF1ARTR	GGCGCATGTCCCTCACA
MYB46_qPCR_F	GAGAGGTGCGGAAAGAGTTG
MYB46_qPCR_R	CTGCAATTTGAGACCACCTG
sgRNA_F1	TGTTTTAGAGCTAGAAATAGCAAGT
gRNA-Ra2	GCACCGACTCGGTGCCAC
