# Supplementary material for: CRISPR-Cas9-mediated deletions of FvMYB46 in Fragaria vesca reveal its role in regulation of fruit set and phenylpropanoid biosynthesis
Source: BMC Plant Biol. 2025 Feb 25;25:256. doi: 10.1186/s12870-024-06041-0 (PMC11853751; doi:10.1186/s12870-024-06041-0)
Supplement: Supplementary file 4 — Supplementary Material 4 [file 12870_2024_6041_MOESM4_ESM.pdf]

Supplementary figure S1.

A

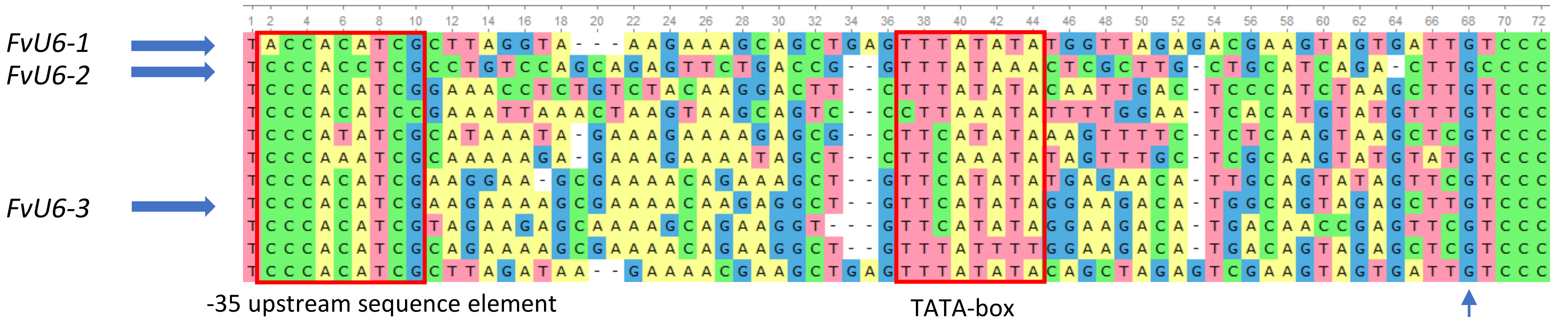

B

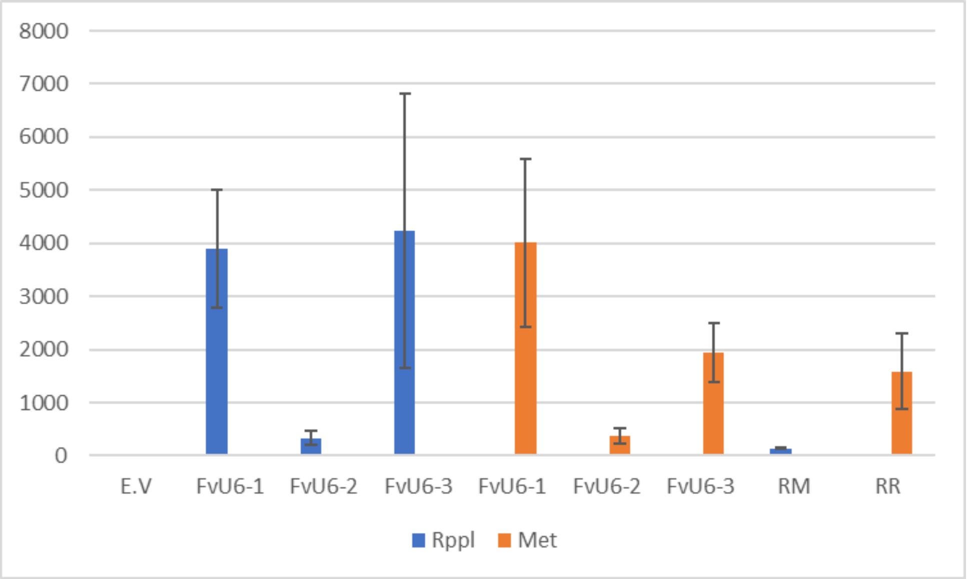

Supplementary figure 1. Identifying endogenous U6-promoters for gRNA expression *in F. vesca*. A) Alignment of the upstream U6-promoter sequence of snRNA genes identified in *F. vesca*. The promoters used for transient expression analyses in transient expression assays is indicated. Blue upwards arrow: transcriptional start site. B) Transient expression analysis of 2 different gRNA (*RPPL1, Met1*) in berries of *F. vesca* using the *FvU6-1, FvU6-2* or *FvU6-3* promoters compared to *AtU6*-promoter (RM, *AtU6-Met1-gRNA*; RR*, AtU6-RPPL1-*gRNA), E.V, Empty vector.

Supplementary figure S2

A

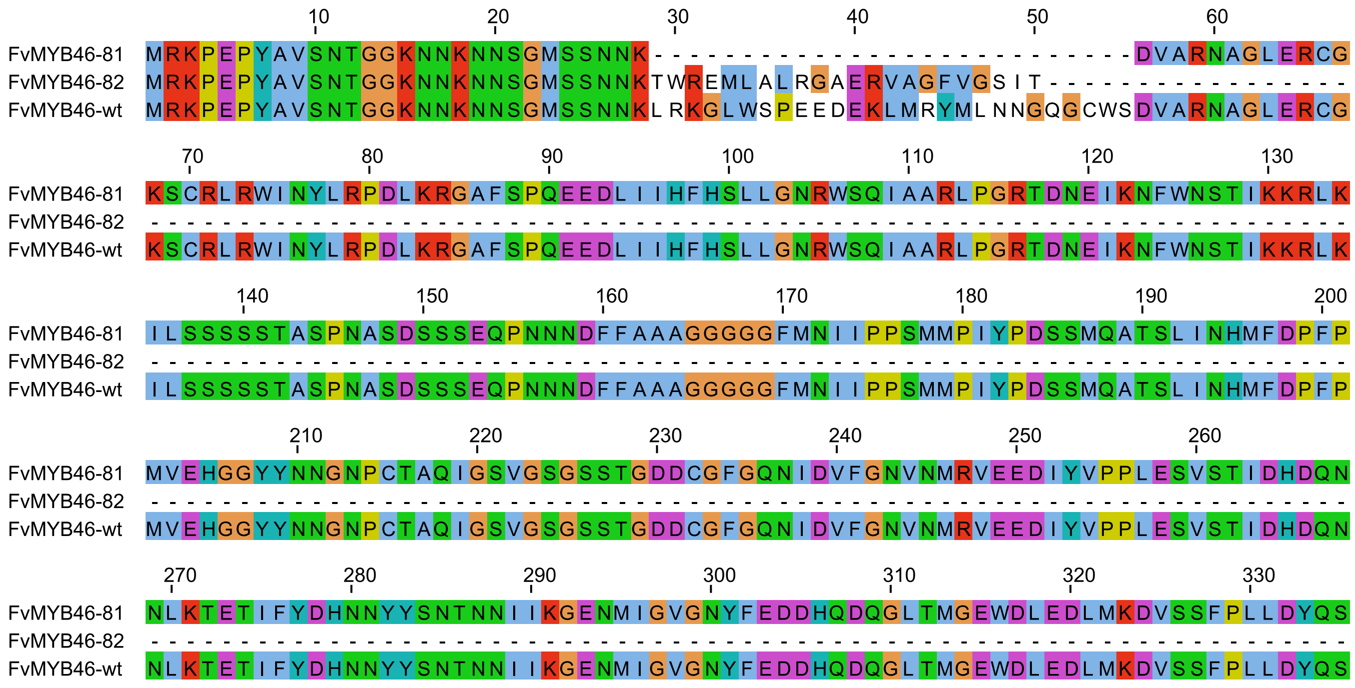

B

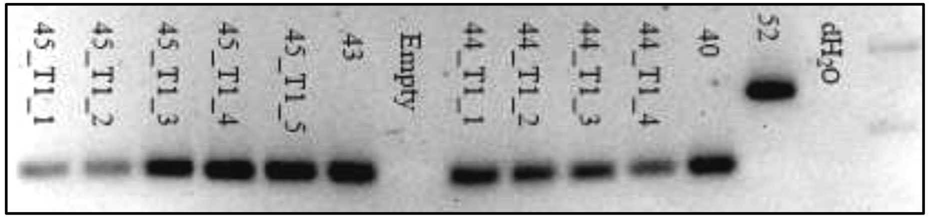

C

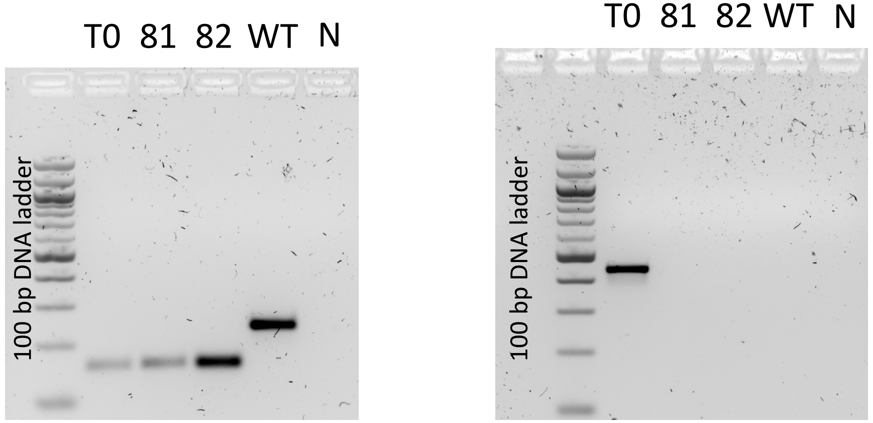

Supplementary figure 2. CRISPR-cas9 mediated deletions of *FvMYB46*. A) Alignment of predicted FvMYB46-81 and FvMYB46-82 translation products with wild type FvMYB46-protein. B) PCR-screening of DNA from T0-plants 40, 43 and 52 (neg) and progeny of 44 and 45-plants with gene specific primers flanking gRNA1 and gRNA2. Progeny of 43 and 44 were renamed *FvMYB46-82* and *FvMYB46-81/82* respectively C) Genotyping of DNA from T0 (44), *FvMYB46-81/82*, *FvMYB46-82* and WT-plants used for transcriptomic and phenotypic studies (left). Genotyping of T0, *FvMYB46-81/82* and *FvMYB46-82* plants with Cas9-specific primers demonstrating no presense of T-DNA in homozygous and biallelic plants used in this study.

Supplementary figure S3

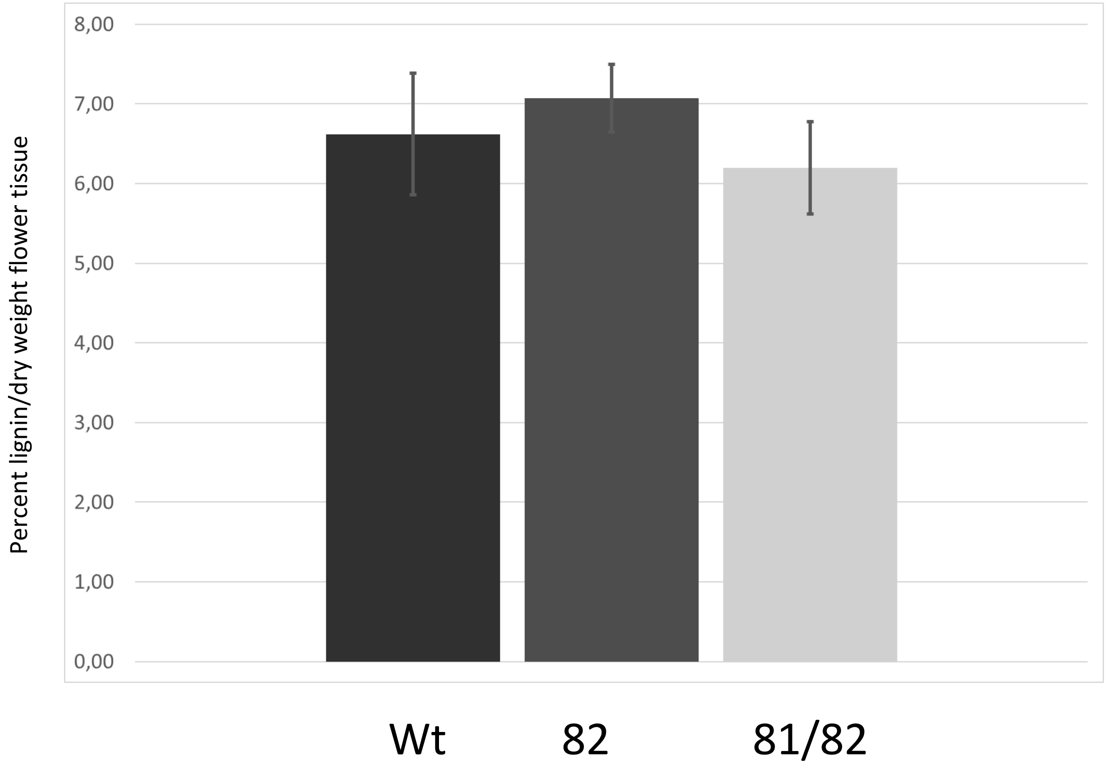

Supplementary figure 3. Lignin content in wt, *FvMYB46-82* and *FvMYB46-81/82* flowers as percent of total dry weight determined by the Klason procedure.

Supplementary figure S4

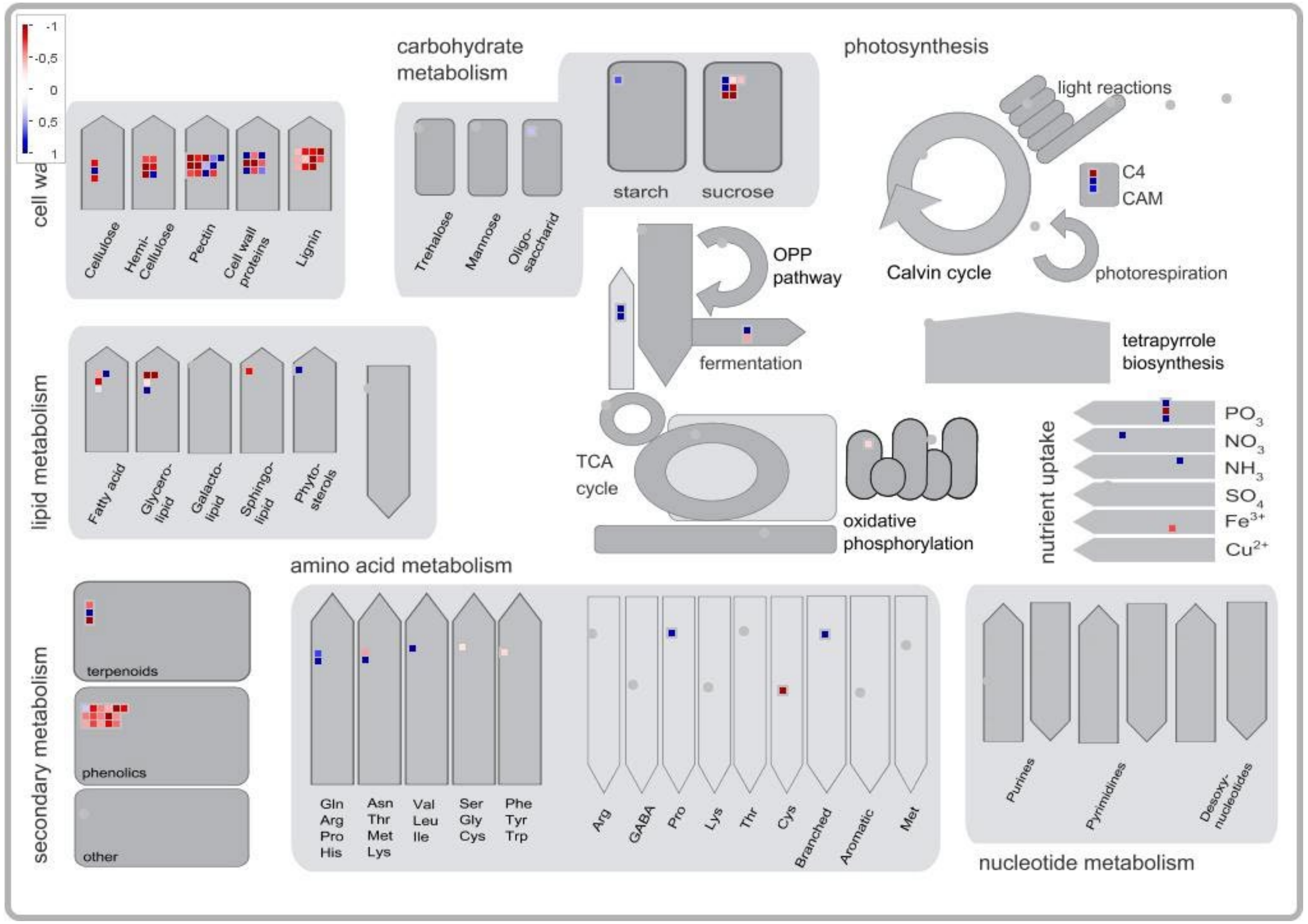

Supplementary figure 4. Visualization of enriched metabolic pathways in the *FvMYB46-82* mutant. Differentially expressed genes (DEGs) in mutant plants compared to wild type displayed onto metabolic pathways using the MAPMAN software for flowers. Blue cells: upregulation *in FvMYB46-82* compared to wild type; red cells: downregulation in *FvMYB46-82* compared to wild type.

Supplementary figure S5

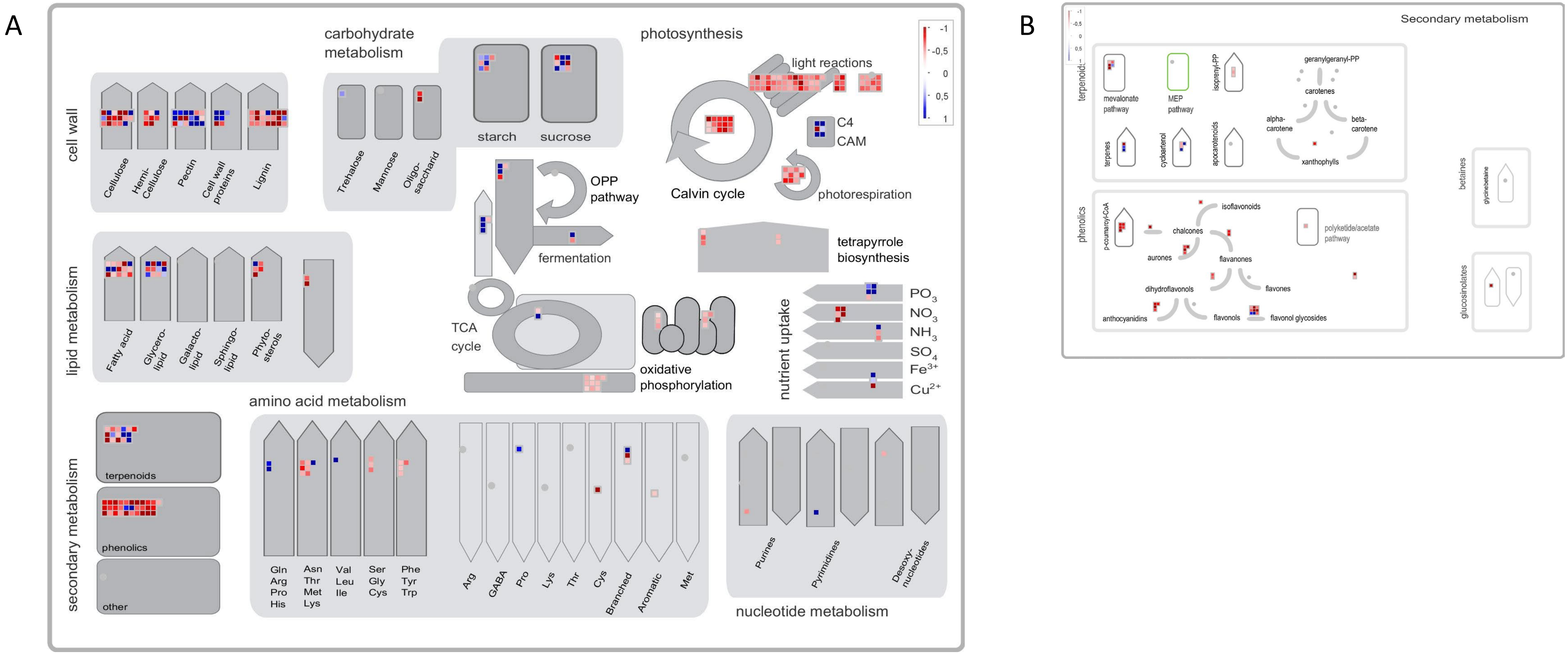

Supplementary figure 5. Visualization of enriched metabolic pathways in the FvMYB46-81/82 mutant. Differentially expressed genes (DEGs) in mutant flowers compared to wild type displayed onto metabolic pathways using the MAPMAN software. A subset of the data are shown in figure 5 C. A) Central metabolic pathways. B)) Secondary metabolism, including phenolics, flavonoid biosynthesis and terpenoids. Blue cells: upregulation in *FvMYB46-81/82* compared to wild type; red cells: downregulation in *FvMYB46-81/82* compared to wild type.

Supplementary figure S6

	Compound	W- flowers	FvMYB46-82-flowers	FvMYB46-81/82-flowers
1	Catechin	0,86±0,09	0,94±0,33	0,57±0,27
2	Proanthocyanin 1	6,23±1,59	5,32±1,97	5,91±0,23
3	Epicatechin	5,17±0,29a	2,19±0,27b	2,69±0,88b
4	Proanthocyanin 2	3,10±0,70	2,69±0,44	2,09±0,76
5	Proanthocyanin 3	20,27±1,38a	19,69±3,09a	12,71±3,65b
6	**Sum Flavan-3-ols**	**35,64±2,70a**	**30,82±5,55ab**	**23,98±4,92b**
7	Coumaric acid-derivative	0,09±0,02	0,10±0.00	0,07±0,01
8	Coumaric acid-derivative	0,18±0,04	0,16±0,03	0,12±0,04
9	Coumaric acid-derivative	0,07±0,02	0,07±0,01	0,05±0,01
10	**Sum Hydroxycinnamic acid derivatives**	**0,34±0,04**	**0,32±0,04**	**0,24±0,07**
11	Ellagic acid - derivative 1	0,30±0,02	0,32±0,02	0,32±0,07
12	Ellagic acid - derivative 2	0,42±0,06a	0,35±0,04a	0,21±0,08b
13	Ellagic acid - derivative 3	15,29±0,18	17.0±1,38	15,94±3,88
14	Ellagic acid - hexoside	2,85±0,17	2,09±0,09	2,25±0,48
15	**Sum Ellagic acid derivatives**	**18,86±0,37**	**19,76±1,53**	**18,72±3,72**
16	Quercetin-3-glucuronide	0,04±0.00	0,06±0,01	0,04±0,01
17	Quercetin-glucoside	6,74±0,44a	5,82±1,27ab	3,87±1,4b
18	Quercetin-3-malonylglucoside	0,07±0,01a	0,11±0,01ab	0,09±0,02b
19	Kaemferol-glucoside	0,24±0,01a	0,19±0,04ab	0,12±0,04b
20	Kaempferol-3-glucuronide	2,51±0,13a	2,13±0,36ab	1,63±0,75b
21	Kaempferol-3-hexoside	0,32±0,02	0,29±0,03	0,22±0,08
22	Kaempferol-3-coumaroylhexoside	0,80±0,12a	0,49±0,05b	0,37±0,17b
23	**Sum flavonols**	**10,72±0,66a**	**9,09±1,70ab**	**6,34±2,43b**

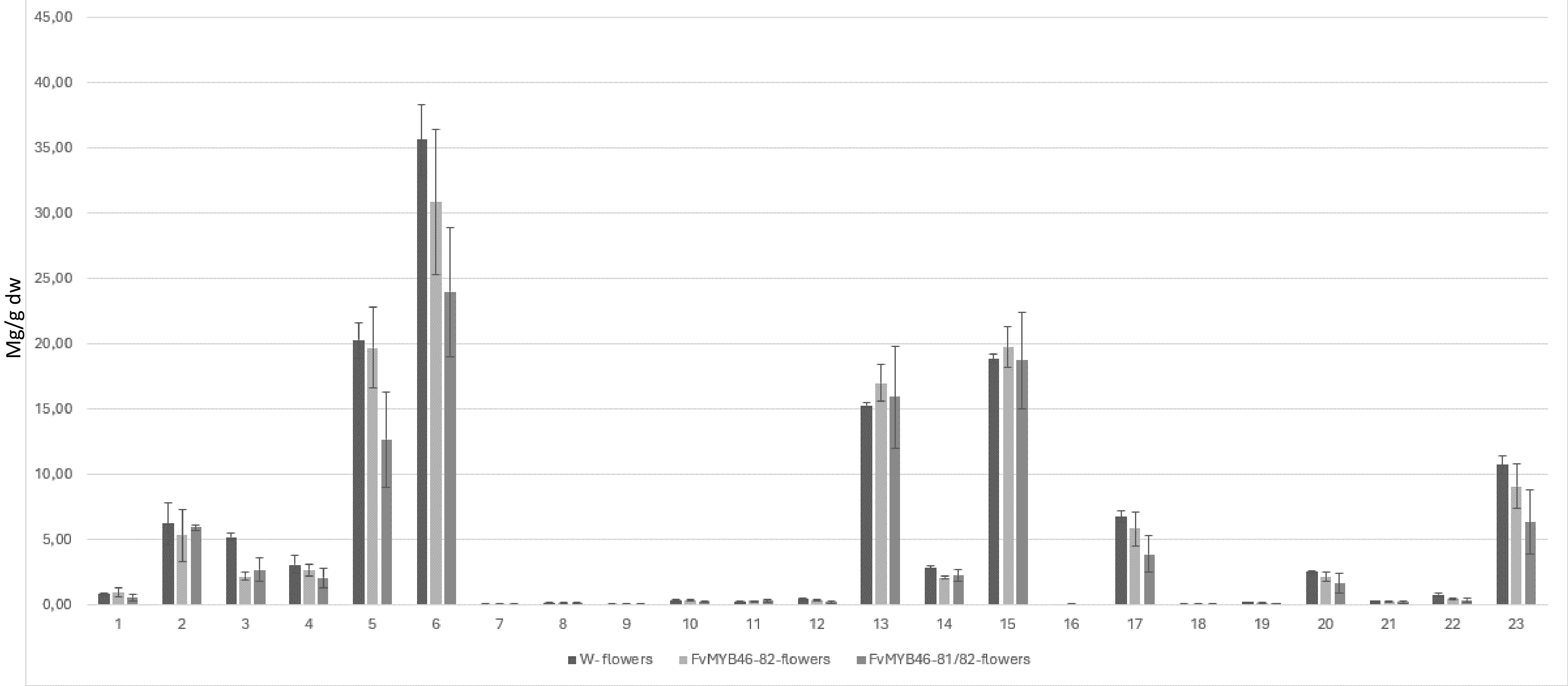

Supplementary figure 6. HPLC analysis of phenolic compounds extracted from flower tissue from wild type and *FvMYB46-deletion* mutants. Individual phenolic compounds were grouped into; flavonols, flavan-3-ols and derivatives of ellagic and hydroxycinnamic acids. Concentrations are presented as mg/g dry weight flower tissue. Values in a row in the table with different letters (a, b) are significantly different ($p < 0.05$) based on Tukey pairwise comparisons test.
